# A novel MOF-based electrochemical sensor for simultaneous quantification of nitrophenols in binary mixtures using artificial neural networks: challenges and opportunities

**DOI:** 10.1039/d5ra03838c

**Published:** 2025-10-29

**Authors:** Abolfazl Kiasadr, S. Maryam Sajjadi, Mehdi Baghayeri

**Affiliations:** a Faculty of Chemistry, Semnan University Semnan Iran; b Department of Chemical Engineering, Lamerd Higher Education Center, Shiraz University of Technology Lamerd Fars Iran sajjadi@sutech.ac.ir (+98) 71 52726630 (+98) 71 52731135; c Department of Chemistry, Faculty of Science, Hakim Sabzevari University Sabzevar Iran

## Abstract

This study introduces an artificial neural network (ANN)-assisted electrochemical strategy for the simultaneous quantification of 2-nitrophenol (2-NP) and 4-nitrophenol (4-NP), two environmentally hazardous pollutants with highly overlapping voltammetric responses. A glassy carbon electrode (GCE) was modified with a multilayer nanocomposite (Ni-MOF-74/Fe_3_O_4_/SiO_2_/NH_2_/β-CD) to enhance mechanical stability and facilitate electron transfer during the oxidation of 2-NP and 4-NP. For the first time, it was demonstrated that the optimal electrocatalytic parameters are concentration-dependent. Therefore, central composite design (CCD) was employed to determine common optimal oxidation conditions across the entire calibration range of both analytes. Despite severe peak overlap and matrix-induced non-linearities, ANN modeling successfully resolved the electrochemical data and provided accurate predictions, yielding high calibration accuracy (*R*^2^ = 0.9302 for 2-NP and 0.9604 for 4-NP) with good reproducibility and broad dynamic range. Considering that the maximum allowable concentration of nitrophenols in environmental samples is 20 ppm, the proposed method offers sufficient sensitivity and a suitable linear range to allow reliable detection and quantification in real environmental matrices. Thus, the integration of ANN with a MOF-based electrochemical sensor provides a novel and robust approach for overcoming signal overlap and non-linear behavior in complex electrochemical systems, offering a promising analytical tool for environmental monitoring of nitrophenols.

## Abstract 2

A sensor based on a Ni-MOF-74/Fe_3_O_4_/β-CD-modified GCE enables simultaneous determination of 2-NP and 4-NP. The data suffers severely overlapping signals and nonlinearity problems, needing to be analyzed by an ANN technique.

## Graphical abstract

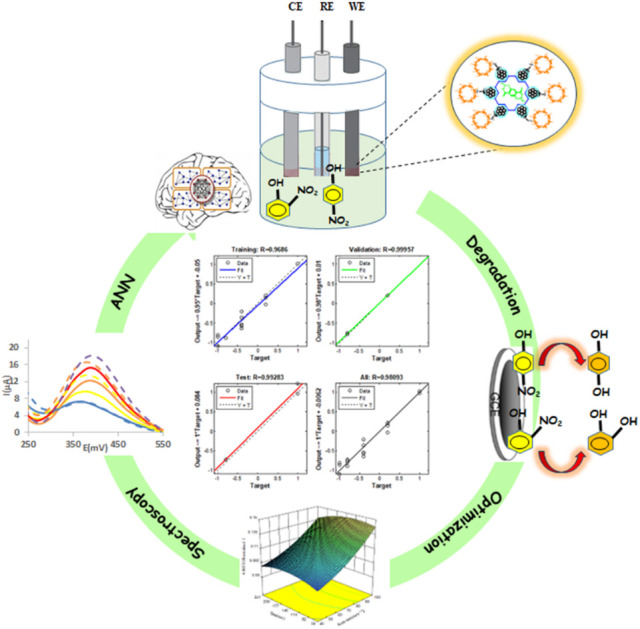

## 1. Introduction

Nitrophenols (NPs) are toxic organic compounds extensively used in synthesizing pesticides, explosives, pharmaceuticals, and dyes.^1,2^ Their widespread industrial application leads to environmental release, where they persist due to high chemical stability, posing risks through toxicity, resistance to biodegradation, and bioaccumulation. According to the US Environmental Protection Agency (EPA) 4-NP and 2-NP are listed among the 126 priority organic pollutants under the Clean Water Act (CWA), with a maximum allowable concentration of 20 ppb in environmental matrices.^3^ Consequently, developing reliable methods for accurately identifying and quantifying NPs in environmental samples is critical to address their ecological and health impacts. Furthermore, simultaneous quantification of 2-NP and 4-NP is particularly important because these compounds often coexist in a variety of matrices owing to their similar structures and properties. Therefore, it is highly required to develop rapid and straightforward analytical techniques that enable the sensitive and selective detection and quantification of 2-NP and 4-NP in their binary mixtures.

Currently, a number of approaches have been established for identifying nitrophenol isomers, such as spectrophotometry, flow injection analysis, capillary electrophoresis, high-performance liquid chromatography, and electrochemical methods.^4–7^ Nevertheless, most of these techniques require preliminary sample preparation steps like separation, extraction, or adsorption, which can be laborious and time-consuming. Compared to other modern methods, electrochemical techniques offer notable benefits, including cost-effective, rapid response, ease of miniaturization, simple operation, and the capability for *in situ* analysis.^6,8^ As a result, substantial research efforts have focused on the electrochemical detection of either 2-NP or 4-NP individually.^9–11^ Yet, achieving their simultaneous determination through electrochemical approaches remains difficult, mainly due to the challenge of overlapping signals.

Obtaining high performance in electrochemical measurements requires careful attention to two key elements: the selection of the measurement technique and the modification of the electrode surface. Various electrochemical strategies, such as potentiometric, amperometric, and voltammetric methods, are available;^12^ among them, anodic stripping voltammetry (ASV) has been recognized as a powerful approach for the detection of trace analytes by combining a preconcentration step with a stripping step, thereby significantly enhancing sensitivity.^13,14^ Additionally, when square-wave voltammetry (SWV) is applied in the stripping process, the resulting square-wave anodic stripping voltammetry (SWASV) method offers numerous advantages over more conventional techniques such as linear sweep voltammetry and differential pulse voltammetry.^15^ In parallel, modification of the electrode surface plays a significant role in improving the sensitivity, selectivity, and stability of electrochemical measurements.^16^ Accordingly, metal–organic frameworks (MOFs) have emerged as materials of considerable promise for electrode surface modification, offering a wide range of structural diversity, adjustable pore sizes, and ease of post-synthetic functionalization, all of which enable the formation of unique interactions between analytes and the electrode interface.^17,18^

In the field of chemical data analysis, two common strategies are recognized: linear and non-linear methods. Linear methodologies comprise multiple linear regression (MLR), principal component regression (PCR), and partial least squares regression (PLS).^19–21^ The non-linear methodologies include artificial neural networks (ANNs), the support vector machine (SVM) algorithm, the self-organizing map (SOM), radial basis function (RBF) neural networks, and multivariate adaptive regression splines (MARS).^22–26^ ANNs, as non-linear learning algorithms, are designed to establish complex mappings between input and output variables, thereby enabling the prediction of unknown outputs based on appropriate input data.

Although numerous studies have demonstrated the application of ANN technique for the analysis of electrochemical data, to the best of our knowledge, there is no report on simultaneous prediction of 2-NP and 4-NP using either linear or non-linear modelling approaches. Additionally, although several modified electrodes based on MOFs have been employed for quantifying 2-NP and 4-NP, the use of a composite combining Ni-MOF74, Fe_3_O_4_/SiO_2_/NH_2_, and β-cyclodextrin (β-CD) for nanoparticle sensor fabrication has not yet been reported.

In this study, we developed a Ni-MOF74/Fe_3_O_4_/SiO_2_/NH_2_/β-CD nanocomposite film through a five-step synthesis process to modify a glassy carbon electrode (GCE) for the simultaneous quantification of 2-NP and 4-NP. The electrochemical properties of the modified electrodes, along with their responses to the analytes, were investigated using square-wave anodic stripping voltammetry (SWASV). Following optimization of the electrochemical conditions, data were collected for binary mixtures of the analytes. As discussed later, severe signal overlap and significant non-linear matrix effects observed in the measurements, it became necessary to apply a non-linear ANN approach for accurate and reliable analysis of the data set.

## 2. Experimental

### 2.1. Chemicals and reagents

The chemical used in this work were as listed: 2-NP(C_6_H_5_NO_3_-2-nitrophenol), 4-NP(C_6_H_5_NO_3_-4-nitrophenol), 2,5-dihydroxyterephthalic acid(DHTA), Ni(NO_3_)_2_·6H_2_O; *N*,*N* dimethylformamide (DMF), ethanol(C_2_H_5_OH), methanol(CH_3_OH), toluene(C_6_H_5_CH_3_), ammonium hydroxide (NH_4_OH), tetraethyl ortho silane)TEOS (3-amino-propyl-triethoxysilane (APTES), sodium hydroxide (NaOH), hydrochloric acid (HCl), nitric acid (HNO_3_), sulfuric acid (H_2_SO_4_), phosphoric acid (H_3_PO_4_), ferrous chloride tetrahydrate (FeCl_2_·4H_2_O), ferric trichloride hexahydrate (FeCl_3_·6H_2_O), β-cyclodextrin (β-CD), potassium chloride (KCl), sodium chloride (NaCl). All chemical used were of analytical grade and obtained from Merck company. Deionized water was applied to prepare all solutions.

The stock solutions of 2-NP and 4-NP (300 ppm) were made by dissolving 0.300 g of 2-NP and 4-NP in 1 L deionized water and then stored in a refrigerator and used for further experiments. An aliquot of the above stock solutions was diluted daily to prepare the working standard solutions. 0.1 M of phosphate buffer solution (PBS) was used as the blank solution. This buffer was prepared from H_3_PO_4_, NaH_2_PO_4_ and Na_2_HPO_4_ and the pH of the solutions were adjusted in the range of 4.00–9.40.

### 2.2. Instrumentation and software

Electrochemical data was recorded with a three-electrode system connected to a Potentiostat/Galvanostat (OrgaFlex 500, Franc). This setup consisted of an Ag/AgCl reference electrode (saturated KCl) electrode, a Pt wire, and a GCE modified with Ni-MOF74 and β-CD as reference electrode, a counter electrode and the working electrode, respectively. GCE electrode was supplied from Azar Electrode Co, Urmia, Iran. Two others were purchased from Metrohm. The pH of solutions was adjusted using a pH meter with a glass combined electrode (PHS-3BWModel), made of Bell, Italy. An ultrasonic water bath (SW3, Switzerland) operating at a frequency of 50/60 kHz was used for sonication. The material was weight by an analytical balance (Shimadzu, Libror AEU-210, Japan).

Fourier transform-infrared (FT-IR) spectrum of the synthesized MOF was recorded using a Shimadzu 8400s spectrometer (Japan) with KBr pressed powder discs. The morphology and size of synthesized nanoparticles were analyzed using a TESCAN MIRA3 LMU field emission scanning electron microscopy (FE-SEM) (Tescan, Brno, Czech Repubic) operated at an acceleration voltage of 15 kV. Elemental composition and mapping of the nanoparticles were analyzed using energy-dispersive X-ray spectroscopy (EDX) with an EDAX detector integrated into the same FE-SEM system. Transmission electron microscopy (TEM) was performed using a Philips CM120 microscope operated at an accelerating voltage of 120 kV.

X-ray diffraction (XRD) analysis was carried out using a Bruker D8 diffractometer (USA) equipped with Cu Kα radiation (*λ* = 1.54051 Å, 35 kV, 15 mA) to determine the chemical structure of the synthesized material. Thermogravimetric analysis (TGA) was performed using a LINSEIS TG/DTA instrument (STA PT 1600, Germany) to assess the thermal stability of the synthesized nano-adsorbent.

All experimental design analyses were conducted using Design-Expert software, trial version 13 (Stat-Ease Inc., Minneapolis, MN), available at (“Stat-Ease, http://www.statease.com/software.html.”, n.d.). For all calculations, Matlab 2024 was used; and neural network toolbox was applied to perform ANN analysis. The voltammogram data was extracted as excel file and then converted to MATLAB format.

### 2.3. Preparation of the Ni-MOF74/Fe_3_O_4_/SiO_2_/NH_2_/β-CD

**Scheme 1:**
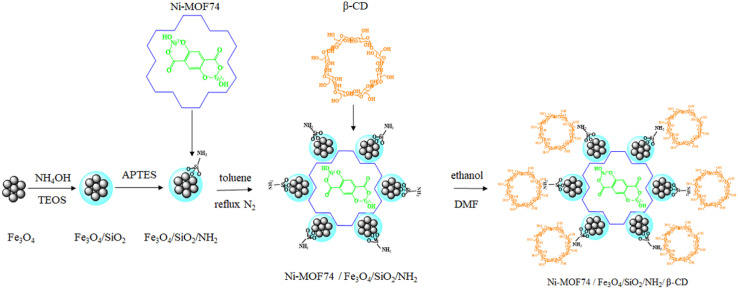
Synthesis procedure of preparing Ni-MOF74/Fe_3_O_4_/SiO_2_/NH_2_/β-CD nanoparticles.

Ni-MOF74/Fe_3_O_4_/SiO_2_/NH_2_/β-CD NPs were synthesized through a five-step process as illustrated in Scheme 1 and described in detail in the following subsections.

#### 2.3.1. Synthesis of Ni-MOF74

Ni-MOF74 NPs were synthesized using hydrothermal method as described in previous report.^27^ First, 0.2179 g of 2,5-dihydroxyterephthalic acid (DHTA) and 1.076 g Ni(NO_3_)_2_·6H_2_O were dissolved in mixed solvent contain DMF, ethanol and deionized water with molar ratio 1 : 1 : 1 and stirred for 30 min at room temperature. Next, the prepared solution was poured into a 100 ml Teflon-lined stainless-steel autoclave and heated in an oven at 120 °C for 24 h to obtain Ni-MOF74 NPs. Afterward, the product was consequently washed out three times with the mixed above solvent. Finally, Ni-MOF74 NPs were dried in a vacuum oven at 80 °C for 5 hours.

#### 2.3.2. Synthesis of Fe_3_O_4_ nanoparticles

Fe_3_O_4_ NPs were synthesized using hydrothermal method as reported in ref. 28 with a little modification. Briefly, a mixture solution of FeCl_3_·6H_2_O (0.04 mol L^−1^) and FeCl_2_·4H_2_O (0.02 mol L^−1^) with mole ratio 2 : 1 was prepared 50 ml of the mixture was sonicated for 5 min at room temperature. Then, 10 ml of NH_4_OH was added into the solution dropwise and sonicated for 30 min. Next, the solution was transferred to the autoclave heated in an oven at 160 °C, for 8 h. Afterward, the obtained Fe_3_O_4_ NPs were accumulated *via* a strong magnet and consecutively washed out three times using ethanol and subsequently washed with deionized water for three times. Eventually, the product was dried in a vacuum oven for 4 hours at 60 °C.

#### 2.3.3. Synthesis of Fe_3_O_4_/SiO_2_/NH_2_

The prepared Fe_3_O_4_ NPs were modified using TEOS and APTES as reported in ref. 29 and 30. Briefly, first, 0.500 g of the NPs was dissolved in 20 ml of ethanol and then sonicated for 15 min at room temperature. Subsequently, 8 ml of TEOS was added dropwise into the suspension and stirred vigorously at room temperature for 24 h. Then, 0.40 ml APTES was added into the above suspension at 60 °C for 24 h. Next, the product was collected using a powerful magnet and washed as the same as that of the above prepared Fe_3_O_4_ NPs. Then the NPs were dried at 60 °C in a vacuum oven for 6 h.

#### 2.3.4. Synthesis of Ni-MOF74/Fe_3_O_4_/SiO_2_/NH_2_

0.500 g of Ni-MOF74, 0.500 g of Fe_3_O_4_/SiO_2_/NH_2_ NPs was dissolved in 20 ml of toluene and sonicated for 15 min at room temperature. Consequently, the suspension was refluxed under N_2_ atmosphere for 3 hours at 110 °C. The product was collected using an external magnetic field, thoroughly washed with ethanol and deionized water, and finally dried under vacuum at 60 °C for 5 hours to obtain the Ni-MOF74/Fe_3_O_4_/SiO_2_/NH_2_ nanoparticles.

#### 2.3.5. Synthesis of Ni-MOF74/Fe_3_O_4_/SiO_2_/NH_2_/β-CD

In this step, the above prepared NPs was modified by β-CD. In this purpose, 0.100 g of Ni-MOF74/Fe_3_O_4_/SiO_2_/NH_2_ and 1.000 g β-CD were dissolved in 20 ml of DMF and sonicated for 15 min at room temperature. Consequently, the mixture has been refluxed under argon atmosphere for 5 h at 60 °C. The product was purified by washing three times with each of the following solvents: ethanol, deionized water and DMF. Then obtained Ni-MOF74/Fe_3_O_4_/SiO_2_/NH_2_/β-CD NPs were dried under vacuum at 60 °C for 5 h.

### 2.4. Fabrication of Ni-MOF74/Fe_3_O_4_/SiO_2_/NH_2_/β-CD/GCE

To prepare the working electrode, the glassy carbon electrode (GCE) was initially polished using a 0.05 μm alumina slurry on a polishing cloth, followed by rinsing with deionized water. Then it was put in an ethanol solution and subsequently dried at room temperature. Afterwards, 3 μL of a suspension containing Ni-MOF74/Fe_3_O_4_/SiO_2_/NH_2_/β-CD nanoparticles (0.0100 g mL^−1^ in ethanol) were drop-cast onto the surface of the GCE and allowed to dry at room temperature.

### 2.5. Experimental procedure

All individual or mixture solutions of 2-NP and 4-NP were prepared in PBSs or acetate buffer medium and the modified electrode was immersed in 10 ml of the solution. The voltammogram of each sample was recorded based on SWASV in the voltage range of −100 to +1000 mV with pulse amplitude 500 mV under potential ASV-1000 mV at a given scan rate and duration time.

## 3. Theory

### 3.1. Response surface methodology and central composite design

Response Surface Methodology (RSM) is a multivariate approach that utilizes mathematical and statistical techniques to model the relationship between a system's output response and its influencing variables.^31^ This method is particularly effective for identifying optimal operating conditions and simultaneously evaluating both individual and interactive effects of variables within a defined experimental range. Commonly used RSM designs include the Box-Behnken Design (BBD) and Central Composite Design (CCD), which enable efficient optimization with a reduced number of experimental runs—thereby minimizing both time and cost.

A central composite design CCD for *k* factors consists of *N* experiments and the total number of experiments (*N*) is given as follows:^32^

1 *N* = 2^*k*^ + 2*k* + *k*_0_

where 2^*k*^ are the experiments from a 2-level factorial design, used to estimate the linear and interaction effects of the factors. And *k*_0_ are repeated experiments at the center point, which provide an estimate of pure experimental error. And 2*k* are the experiments positioned along the axes at a distance *α* from the center, as shown in subsequent equations (eqn (2)). These points establish new extreme values (high/low) for the factors, enabling the measurement of curvature in the model. *α* can be calculated as in the following equation:2
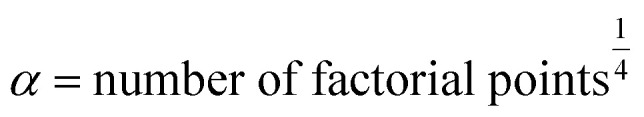


CCD data is analyzed using RSM strategy to establish the relationship between the response variable and influencing factors based on empirical quadratic model, as expressed in the following equation:3
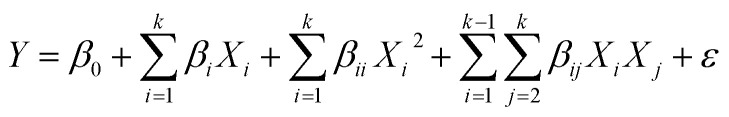
where *Y* represents the response, *X*_*i*_ denotes the factors and *β*_0_ is the intercept term. The coefficients *β*_*i*_ and *β*_*ii*_ corresponded to the linear and quadratic effects of the ith factor respectively; *β*_*ij*_ represents the interaction effect between *X*_*i*_ and *X*_*j*_, and *ε* is the residual error term. The adequacy of the model is assessed through the analysis of variance (AVOVA), where the model's reliability is evaluated using the coefficient of determination (*R*^2^) and the adjusted *R*^2^ (Adj-*R*^2^). At a 95% confidence level, the model is considered statistically significant when *p*-value of the model is less than 0.05 and *p*-value of lack of fit is more than 0.05.

The adequacy of the fitted model is evaluated through analysis of variance (ANOVA), where the model's reliability is assessed using the coefficient of determination (*R*^2^) and the adjusted *R*^2^. At a 95% confidence level, the model is considered statistically significant when the *p*-value for the model is less than 0.05 and *p*-value of lack-of-fit is more than 0.05.

### 3.2. Artificial neural network

ANNs are considered as promising tools among the recent trends of modeling strategies for nonlinear calibration or classification aims. As a subset of machine learning, ANNs imitates the functioning of biological neuronal systems. An ANN consists of interconnected artificial neurons or nodes that acts as simple processing units, *i.e.* neurons. These nodes are linked through artificial synapses, which exist among both input neurons layer and hidden ones; or hidden neurons layers and output neurons layer, known as weight (*W*_ij_). In a node, input data is processed as follows:4
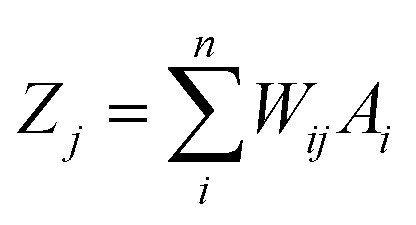
Here, *Z*_*j*_ refers to the output value of *j*th hidden node and *W*_ij_ denotes the weight assigned to the connection between the ith input node to the *j*th hidden one. *A*_*i*_ is the normalized value of the *i*th value of the input variable.

In the ANN algorithm, both input and output data are scaled in the value range of −1 to +1 as described by the following equation (eqn (5)):^22^5
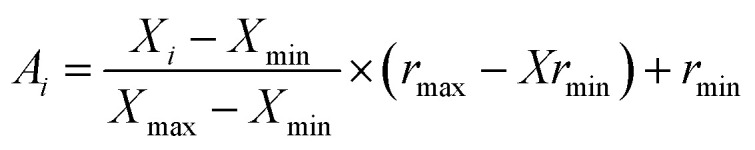
Here, *X*_*i*_ is an *i*th actual input data, *A*_*i*_ is the normalized amount of *X*_*i*_; *X*_min_ and *X*_max_ are the minimum and maximum values of *X*_*i*_; and *r*_min_ and *r*_max_ are assigned to the limits of the coded range in which *X*_*i*_ is scaled.

One of the most widely used ANN algorithm is the back-propagate feed-forward neural network (BPFF-ANN), which was applied in this study to develop a nonlinear calibration curve of the analytes.^33–36^ In the BPFF-ANN method, the weights must be renewed in each iteration until the difference between the experimental output data and the model's predicted values is minimized. The following eqn (6) illustrates the weight update process in each iteration:

6 Δ*W*_*ij*_ = *η* (*t* − *o*)In_*i*_

Here, *t* and *o* represent the actual experimental and predicted output values for each sample, respectively. The weight updates are controlled by *η*, called as the learning factor, in each iteration. The amount of *η* is set to a value smaller than 0.1 and gradually decreases, reducing its influence as the number of iterations increases.

In the BPFF-ANN strategy, the input values for the neurons are obtained from the real world, weighted, and used as input for the first hidden layer. Each subsequent layer receives weighted outputs from the preceding hidden layer. Moreover, the values of output layer come from the real world.

A node in either the hidden or output layer performs two main tasks: first, it computes the weighted sum of the inputs from multiple connections along with a bias value, then applies a transfer function to this sum. Second, it transmits the resulting value through outgoing connections to every node in the next layer, where the same process is repeated.^22^

The number of nodes in the input and output layers corresponds to the number of independent and dependent variables, respectively. In this work, the independent variables are the currents of samples at selected potentials while the dependent variable is the concentration of 2-NP or 4-NP. The network is trained to establish the relationships between the independent and dependent variables by iteratively comparing the predicted and actual concentrations. During the iteration process, the weight matrix and bias vector of each layer are adjusted by a back propagation training algorithm. To reduce the likelihood of converging to a local minimum, ANN analysis is performed under multiple random initializations of the weights.

## 4. Result and discussion

The synthesized Ni-MOF74/Fe_3_O_4_/SiO_2_/NH_2_/β-CD was characterized by FE-SEM, TEM, EDX, XRD pattern and TGA analysis techniques. Moreover, the surface chemistry of different prepared NPs was studied in synthesis steps using FT-IR method to confirms that the modifier has been synthesized successfully as discussed below.

### 4.1. Characterization of Ni-MOF74/Fe_3_O_4_/SiO_2_/NH_2_/β-CD

#### 4.1.1. FT-IR spectra

**Fig. 1:**
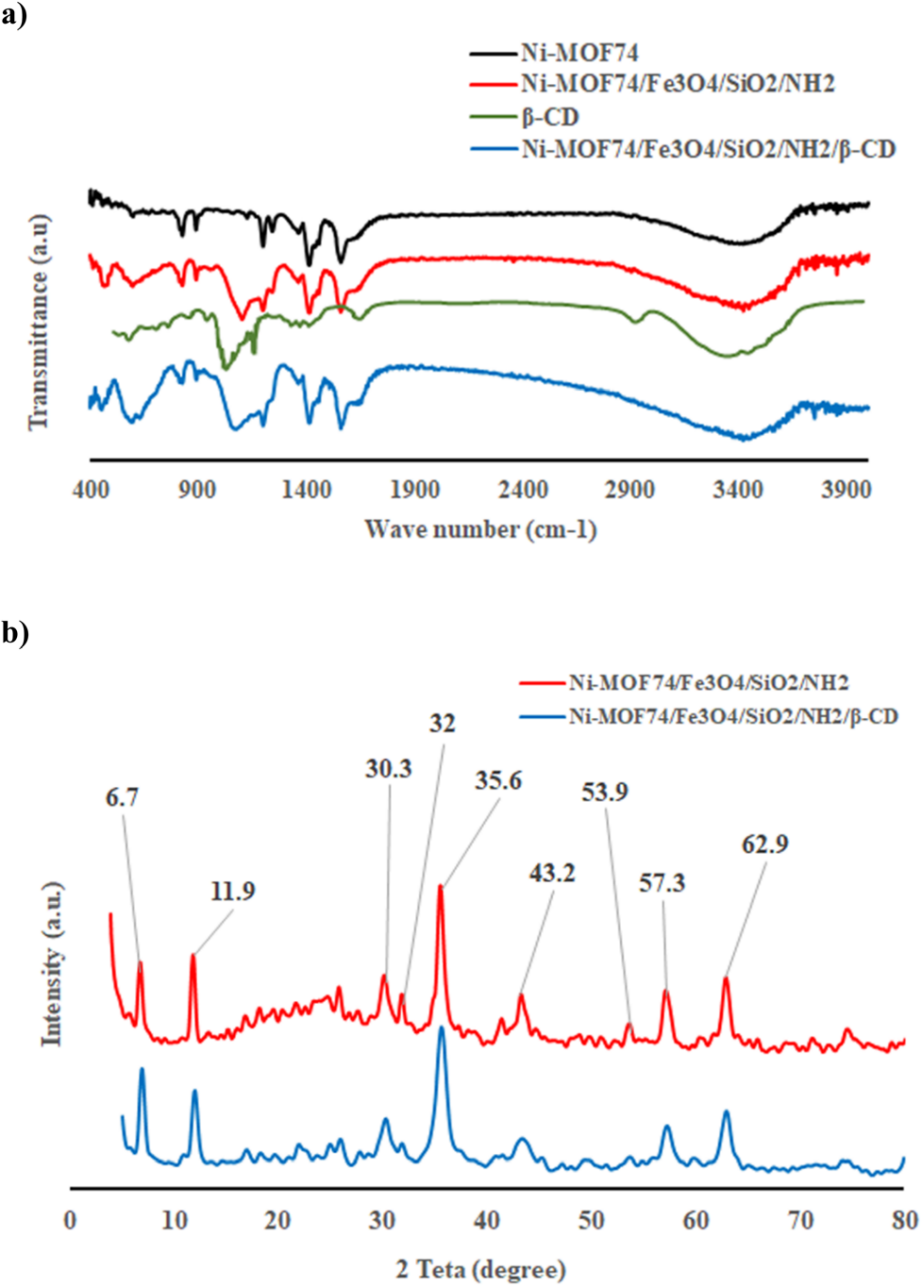
(a) FT-IR spectra of as-synthesized Ni-MOF74, Ni-MOF74/Fe_3_O_4_/SiO_2_/NH_2_, Ni-MOF74/Fe_3_O_4_/SiO_2_/NH_2_/β-CD. (b) XRD powder pattern (inset shows the crystal structure of Ni-MOF74/Fe_3_O_4_/SiO_2_/NH_2_ and Ni-MOF74/Fe_3_O_4_/SiO_2_/NH_2_/β-CD).

The FT-IR spectra of Ni-MOF74 NPs (Fig. 1a), Ni-MOF74/Fe_3_O_4_/SiO_2_/NH_2_ NPs (Fig. 1a) and Ni-MOF74/Fe_3_O_4_/SiO_2_/NH_2_/β-CD NPs (Fig. 1a) were collected over the range of 4000–500 cm^−1^ to confirm the successful surface functionalization of Ni-MOF74 NPs. FT-IR spectrum of Ni-MOF74 NPs (Fig. 1a) shows the broad band at 3300–3500 cm^−1^ assigned to OH stretching vibrations due to the pore-filled water molecules.^37^

Some noticeable bands in the wavenumber range about 1600 to 800 cm^−1^ are attributed to the stretching vibration of the aromatic ring, as existed in DHTA.^38^ Two prominent peaks at 1411 cm^−1^ and 1560 cm^−1^ are assigned to the symmetry and asymmetric vibration of –COO^−^, as present in DHTA, which are coordinated to Ni^2+^, respectively.^39^

The vibration of C–H on the benzene ring was observed in 584 cm^−1^.^37^ The peak at 1242 cm^−1^ is corresponded to the stretching vibration of C–N which maybe because of the adsorption of DMF on the surface of the synthesized MOF.^37^ The intense peaks at 889.1 cm^−1^ and 825.48 cm^−1^ are attributed to Ni–O vibrations.^39^

As shown in Fig. 1a, all characteristics bands for Ni-MOF74 NPs are appeared in the FT-IR spectrum of Ni-MOF74/Fe_3_O_4_/SiO_2_/NH_2_ NPs, asserting the successful modification surface of Ni-MOF74 NPs by Fe_3_O_4_/SiO_2_/NH_2_. Additionally, two observed peaks at 470 cm^−1^ and 1101 cm^−1^ are corresponded to Si–O–Si bending vibrations, which indicates that the Ni-MOF74 NPs are successfully coated by silica.^40^

The prominent peak at 595 cm^−1^ is attributed to Fe–O stretching vibration.^41^ The observed band at 1624 cm^−1^ is attributed to N–H vibration, which is presence in APTES.^42^ FT-IR spectrum of β-cyclodextrin shows a prominent broad band at 3364 cm^−1^ ascribed to O–H stretching mode of β-cyclodextrin. Moreover, there are several noticeable peaks in this spectrum as follows: 1028 cm^−1^ (C–O–C stretching vibration); 1650 cm^−1^ (C–C stretching vibration), 2922 cm^−1^ (C–C stretching vibration), and 3364 cm^−1^ (O–H stretching vibrations).^43^ All of these peaks are characteristic peaks of β-cyclodextrin.

In Fig. 1a, interestingly enough, all characteristics band or peaks for Ni-MOF74/Fe_3_O_4_/SiO_2_/NH_2_ were observed with a little shift in Ni-MOF74/Fe_3_O_4_/SiO_2_/NH_2_/β-CD. Compared to FT-IR spectrum of Ni-MOF74/Fe_3_O_4_/SiO_2_/NH_2_, a blue shift of three bands was observed as follows: Fe–O peak from 595 cm^−1^ to 586 cm^−1^; Si–O–Si strong peak from 1103 cm^−1^ to 1068 cm^−1^; Si–O–Si peak at 470 cm^−1^ to 447 cm^−1^. All of these shifts demonstrate the successful modification of the Fe_3_O_4_ and SiO_2_ groups the synthesized nano-composite.^29^ Moreover, two characteristic peaks of β-CD at 2922 cm^−1^ and 3364 cm^−1^ were broadened and overlapped into a single broad band in the FT-IR spectrum of Ni-MOF74/Fe_3_O_4_/SiO_2_/NH_2_/β-CD (Fig. 1a), corresponded to hydrogen bonding and other interaction forces, asserting the successful modification of β-CD on the surface of Ni-MOF74/Fe_3_O_4_/SiO_2_/NH_2_.

#### 4.1.2. XRD analysis

The structure and crystallinity of Ni-MOF74/Fe_3_O_4_/SiO_2_/NH_2_ and Ni-MOF74/Fe_3_O_4_/SiO_2_/NH_2_/β-CD were characterized by X-ray diffraction (XRD) as shown in Fig. 1b.

In XRD patterns, three characteristic peaks at 2θ values of 6.7°(110), 11.9°(300), and 32° (101) display the similar relative intensities and positions to those reported for Ni-MOF-74, asserting successful incorporation of the MOF structure.^39^

Moreover, distinct peaks at 2*θ* = 30.3 (220), 35.6 (311), 43.2 (400), 53.9 (422), 57.3 (333), 62.9 (440) exhibit a phase face centered cubic structure for Fe_3_O_4_ core, indicating the presence of magnetic nanoparticles.^30^ A broader hump observed between 2*θ* = 20° and 25° suggests the presence of mesoporous silica oxide shell.^44^ Moreover, the diffraction peak for surface–NH_2_ groups on the prepared composite exists at 2*θ* = 20°, which has overlapping with the broad hump for SiO_2_ shell.^45^

The XRD pattern of final product (Fig. 1b) shows less intense hump between 2*θ* = 20° and 25° confirming the successful surface modification of Ni-MOF74/Fe_3_O_4_/SiO_2_/NH_2_ with β-CD.

#### 4.1.3. TGA analysis

**Fig. 2:**
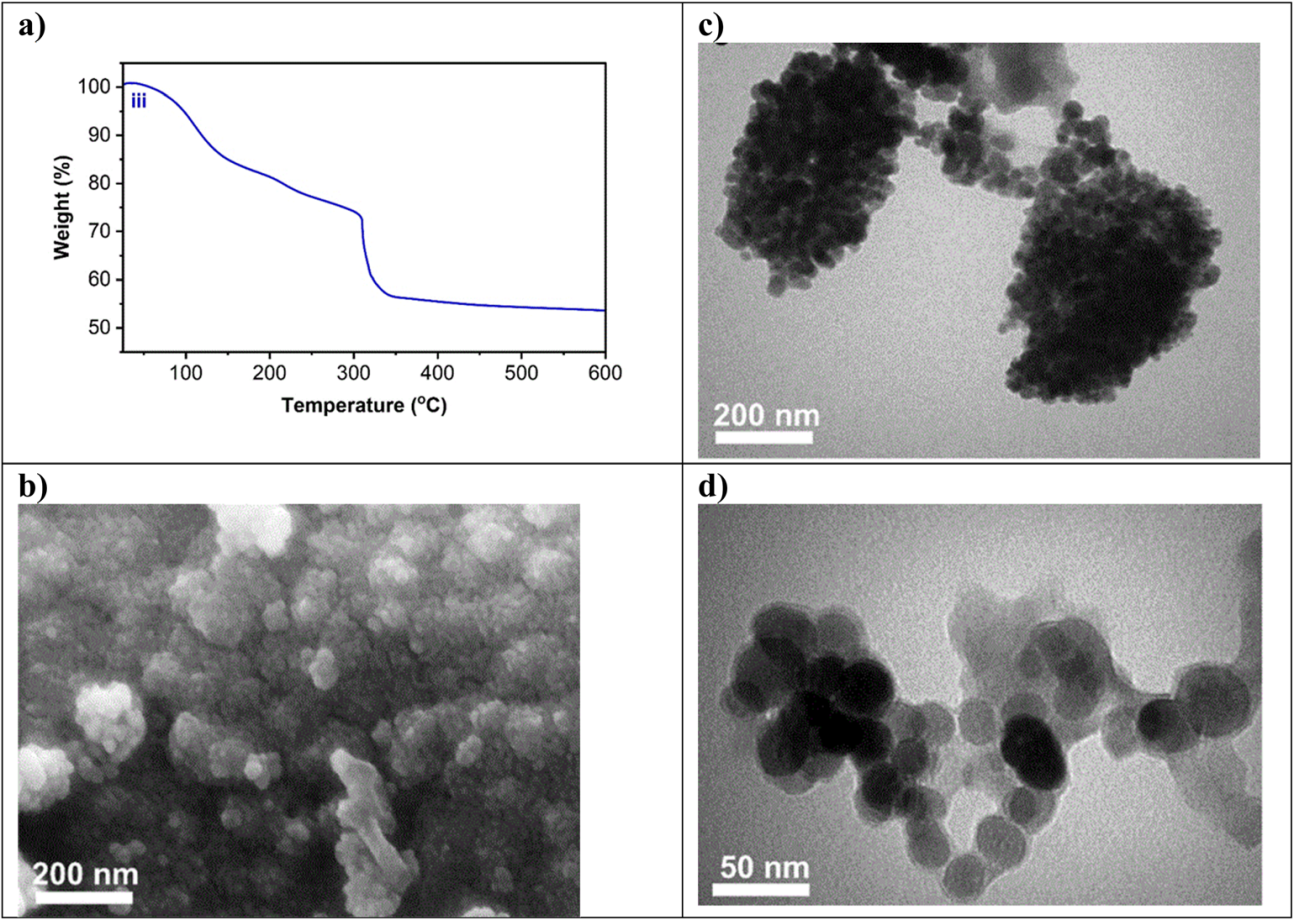
Characterization of Ni-MOF74/Fe_3_O_4_/SiO_2_/NH_2_/β-CD: (a) TGA plot (b) FE-SEM images (inset shows the magnified image), (c and d) TEM images.

TGA analysis was performed to evaluate the amount of organic compounds functionalized on the surface of Ni-MOF74/Fe_3_O_4_/SiO_2_/NH_2_/β-CD (Fig. 2a). The first weight loss observed in the TGA curve (Fig. 2a) occurs at a temperature of about 90 °C, which is attributed to the loss of water molecules,^46^ while the second weight loss at 140 °C is due to evaporation of adsorbed solvents such as internal water.^46^ A weight loss of 4% observed at the third and fourth stage between 224 °C and 342 °C is attributed to the decomposition of β-CD, occurring after the melting of its glucose units.^47^ This corresponds to an estimated β-CD loading of approximately 4 weight% on the synthesized nanocomposite. The weak weight loss at temperature higher than 342 °C could be attributed to the destruction of the structure MOF^37,48^ and to the oxidation of Fe_3_O_4_ to Fe_2_O_3_.^49^

#### 4.1.4. FE-SEM and TEM patterns

FE-SEM was used to investigate surface morphology and structure of the synthesized Ni-MOF74/Fe_3_O_4_/SiO_2_/NH_2_/β-CD composite. The FE-SEM image (Fig. 2b) reveals that the NPs exhibit a uniform morphology with quasi-spherical in shape.

TEM image of Ni-MOF74/Fe_3_O_4_/SiO_2_/NH_2_/β-CD is shown in Fig. 2c and d exhibiting the NPs are well dispersed with 50 nm of average size. In this image, the dark and non-spherical parts could be assigned to Ni-MOF74 and the spherical parts could be attributed to Fe_3_O_4_ magnetic nanoparticles.^50^

#### 4.1.5. EDX analysis

**Fig. 3:**
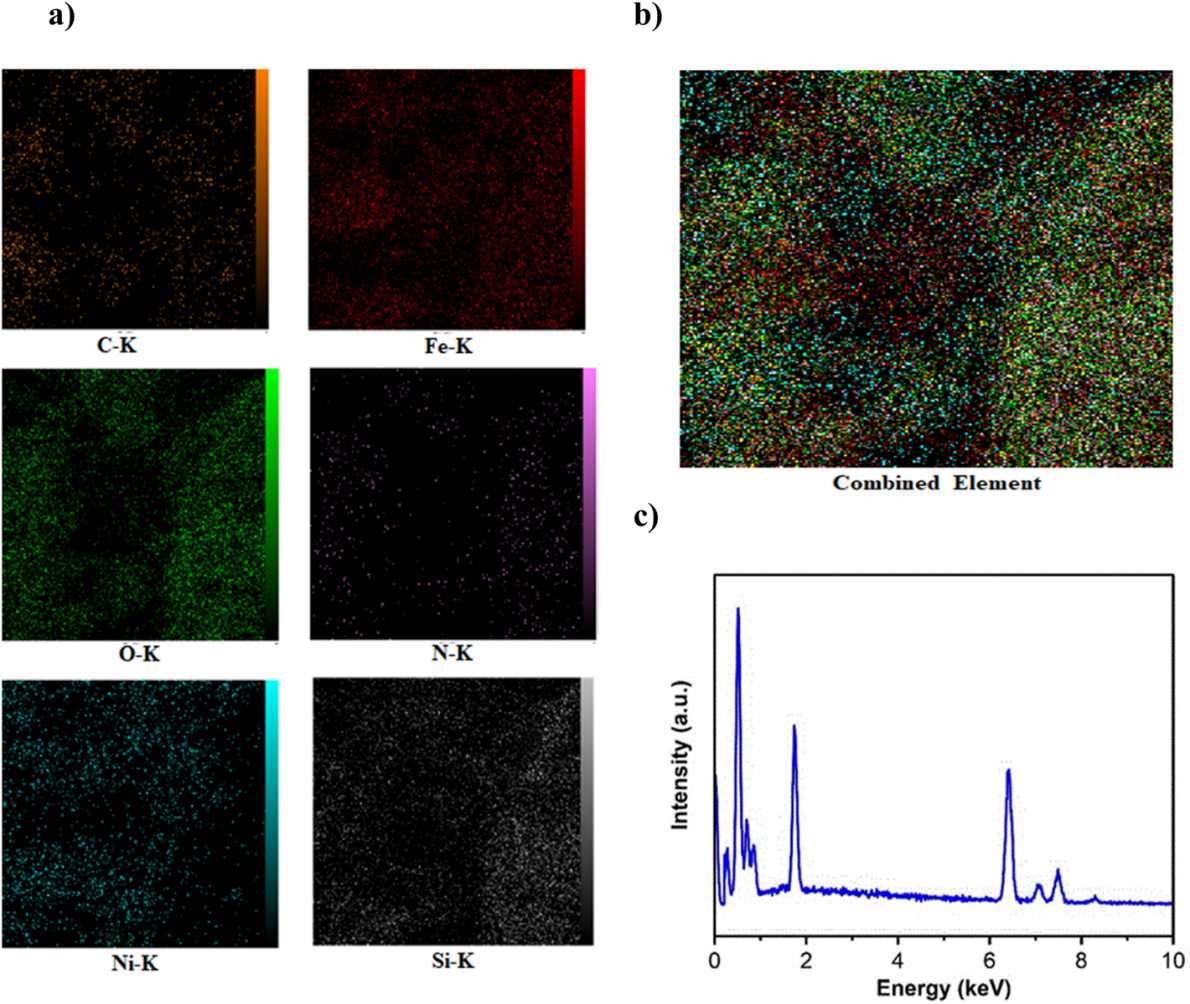
EDX images of Ni-MOF74/Fe_3_O_4_/SiO_2_/NH_2_/β-CD: (a) distribution of each involving element in the nanocomposite (indicated by different colors); (b) distribution for the combined elements; (c) EDX spectrum of the nanocomposite.

The mapping of each element using EDX analysis (Fig. 3a) in a sample of Ni-MOF74/Fe_3_O_4_/SiO_2_/NH_2_/β-CD shows that all elements are spread out within the area of the crystalline particles. The combined elements map (Fig. 3b) and EDX spectrum (Fig. 3c) shows a uniform elemental distribution with full coverage of C, N, Ni, O, Si, Fe particles on the surface of nanocomposite, indicating the successful incorporation of all expected components in Ni-MOF74/Fe_3_O_4_/SiO_2_/NH_2_/β-CD NPs.

#### 4.1.6. Reliability in modified electrode performances

Reliability of Ni-MOF74/Fe_3_O_4_/SiO_2_/NH_2_/β-CD/GCE performance was evaluated through reproducibility tests. For each analyte (2-NP and 4-NP), measurements were conducted at four concentration levels, with three replicates per level. Prior to each experiment, a freshly prepared modified electrode was used. Voltammograms were recorded under the following conditions: pH 9.4, scan rate 70 mV s^−1^, and duration time of 145 s. The corresponding RSD values are presented in Table 1. The results confirmed that the Ni-MOF74/Fe_3_O_4_/SiO_2_/NH_2_/β-CD/GCE exhibited satisfactory reproducibility for 2-NP (3.6–5.7%) and 4-NP (3–11%) at all tested concentration levels.

**Table 1:** RSD (%) of synthesized modified electrode for 2-NP and 4-NP (*n* = 3)

RSD (%) conc. (ppm)	2-NP	4-NP
10	3.6	5.1
30	5.6	7.9
60	4.5	3
100	5.7	11

### 4.2. Procedure optimization

In order to achieve the optimal electrocatalytic performance of Ni-MOF74/Fe_3_O_4_/SiO_2_/NH_2_/β-CD/GCE towards the 2-NP and 4-NP oxidation, the experimental factors including buffer type, pH of solution, scan rate and duration time were investigated. In this work, buffer type and pH were optimized using ‘one-variable-at-a-time’ method at a constant value of other factors such as concentration of analytes, scan rate and duration time. Then at fix optimal condition of buffer type and pH, the other factors were investigated based on statistical experimental design strategy. In all experiments, the voltammogram of modified electrode was recorded, as described in Section 2.5 and then the anodic peak current of the analytes was used as response for further analysis. However, the results showed that the background of voltammogram is not the same for all experiments; moreover, the recorded data was noisy. Therefore, prior to analysis, the obtained data was preprocesses as described below.

#### 4.2.1. Optimization of buffer type and pH and preprocessing of voltammograms

**Fig. 4:**
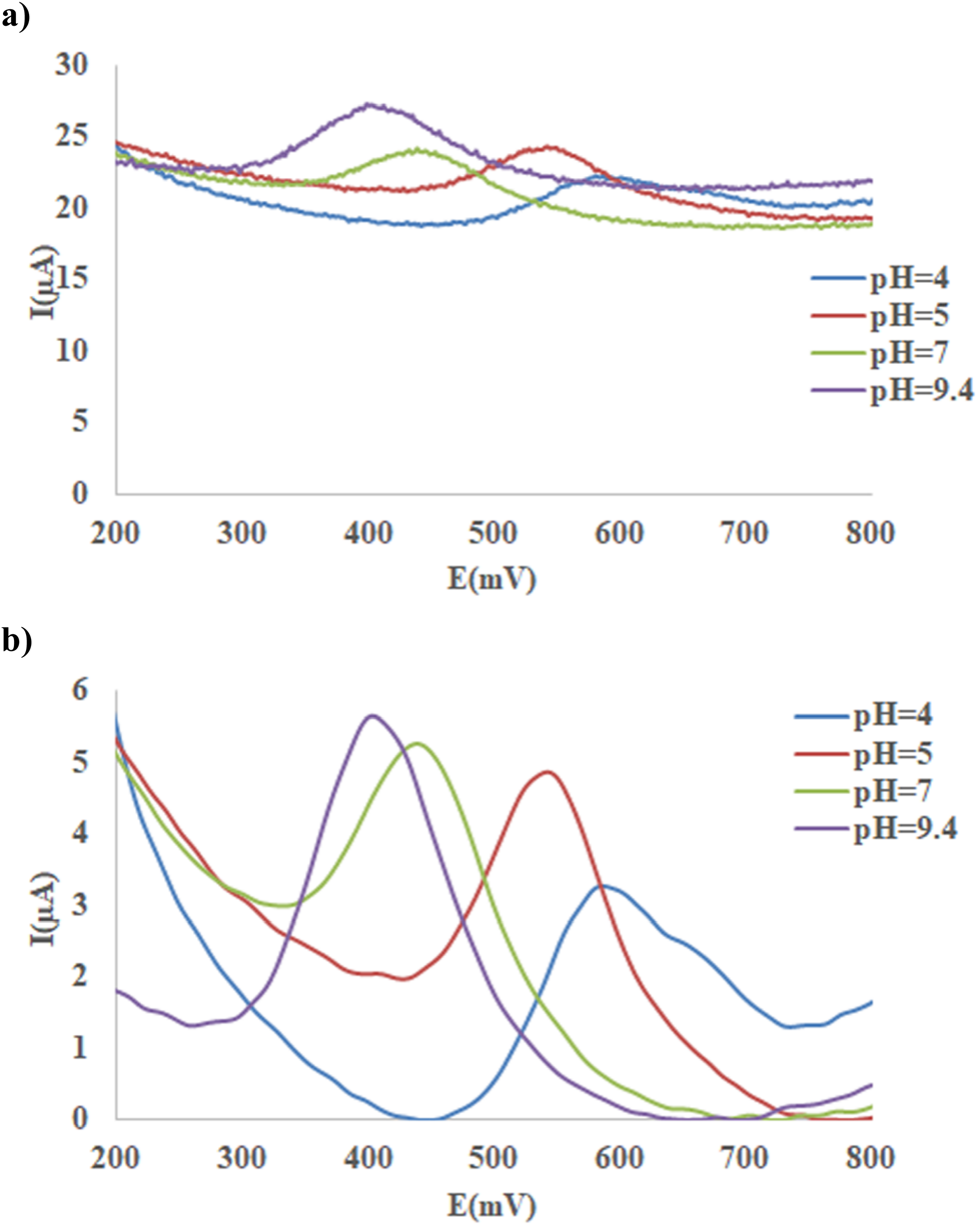
SWAS voltammograms of 4-NP (30 ppm) at the Ni-MOF74/Fe_3_O_4_/SiO_2_/NH_2_/β-CD/GCE surface in the PBS (0.1 M) at different pH (4.00–9.40), scan rate 100 mv s^−1^,duration time120 s, pulse = 500 mV. (a) Experimental data, (b) pretreated data.

Fig. 4a shows a set of SWASV of Ni-MOF74/Fe_3_O_4_/SiO_2_/NH_2_/β-CD/GCE in PBS (0.1 M) and 30 ppm 4-NP at scan rate 100 mv s^−1^,duration time120 s, pulse = 500 mV and pH values ranging between 4.00-9.40. To pre-process this data, first, the minimum of signal of each voltammogram was subtracted from the voltammogram and then smoothed using Savitzky–Golay algorithm (Fig. 4b).^51^ The same data was collected for 4-NP (30 ppm) in acetate buffer at the above condition and the results showed, the maximal anodic peak for acetate media was smaller than that of PBS. Similarly, the performance Ni-MOF74/Fe_3_O_4_/SiO_2_/NH_2_/β-CD/GCE was also investigated for oxidation of 30 ppm of 2-NP at the same procedure describe above for 4-NP (Fig. S1) and the results revealed the maximum signal of 2-NP was obtained in PBS (0.1 M) at 9.40 of pH and this optimal condition is the same as that of 4-NP.

**Scheme 2:**
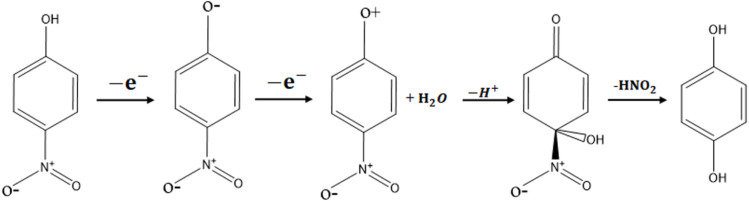
Possible 4-NP oxidation reaction mechanism.

It was deduced from Fig. 4, the highest anodic peak current was observed at pH 9.40, which was therefore chosen as the optimal condition. Previous studies^52,53^ suggest that 4-NP oxidation begins with its deprotonation, producing nitrophenoxy cations that are highly reactive and may couple to form polymers or undergo further chemical transformations. These transformations can involve nitro group elimination or substitution by hydroxyl groups, yielding non-nitrogenated phenolic or quinonic intermediates (Scheme 2). A comparable mechanism is expected for 2-NP. Therefore, at pH 9.4, nitrophenols predominantly exist in their deprotonated state, enabling an initial reaction step to proceed without electron transfer, thereby accelerating the oxidation process.

The other two main variables influencing the measurement 2-NP and 4-NP are as follows: scan rate (*A*) duration time (*B*). In this study, these parameters were optimized using an experimental design approach as described in the following sections.

#### 4.2.2. Central composite design

After conducting many preliminary experiments, we figure out that the optimal condition of scan rate and duration time are highly dependent on the concentration of 2-NP or 4-NP in the solution. Due to the intrinsic property of calibration purposes, *i.e.*, the concentration of analyte changes at different samples to obtain the calibration curve, we proposed to optimize these two main parameters for each concentration of analyte used in the calibration samples and then achieve a common optimal condition for all concentration based on desirability function. Indeed, in electrochemical analysis, the dependency of concentration of analyte on the experimental condition has been a neglected issue.

Scan rate and duration time, as the main factors affecting the quantification 2-NP or 4-NP, were investigated based on CCD design at 9.40 of pH in PBS (0.1 M) as deduced from the above optimization procedure. It should be mentioned that the calibration set of each analyte was composed of four samples with concentration 10 ppm, 30 ppm, 60 ppm and 100 ppm; and the same CCD design was used for each sample.

According to CCD design, five levels of each factor are defined, which are reported in Table 2 based on the real and coded levels as follows: −*α*, −1, 0, 1, +*α*. Overall, different combinations of these levels of the factors lead to a design with eleven experiments (Table 1).

Experimental conditions for central composite design and experimental responses for the analytes in PBS 0.1 M, pH = 9.40 media

**Table:** 

Factors	Levels				
	−*α*	−1	0	+1	+*α*
A: Scan rate(mv s^−1^)	27.5	40	70	100	112
B: Duration time (sec)	11	50	145	240	280

**Table:** 

Run	Factors					Anodic peak current				
	*A*	*B*	*Y*_1-2-NP10_	*Y*_2-4-NP10_	*Y*_1-2-NP30_	*Y*_2-4-NP30_	*Y*_1-2-NP60_	*Y*_2-4-NP60_	*Y*_1-2-NP100_	*Y*_2-4-NP100_
1	70	11	0.162	0.102	0.129	0.092	0.145	0.099	0.120	0.089
2	112	145	0.200	0.171	0.190	0.176	0.197	0.132	0.143	0.095
3	70	145	0.212	0.178	0.131	0.109	0.133	0.089	0.112	0.062
4	70	280	0.224	0.135	0.154	0.083	0.137	0.092	0.095	0.072
5	70	145	0.185	0.078	0.131	0.092	0.136	0.086	0.097	0.083
6	100	50	0.203	0.152	0.195	0.148	0.164	0.122	0.151	0.106
7	40	240	0.053	0.117	0.074	0.092	0.085	0.067	0.064	0.059
8	70	145	0.126	0.102	0.145	0.134	0.177	0.111	0.119	0.066
9	40	50	0.066	0.094	0.100	0.086	0.111	0.074	0.069	0.072
10	27.5	145	0.043	0.079	0.073	0.070	0.085	0.058	0.059	0.052
11	100	240	0.246	0.164	0.193	0.141	0.175	0.103	0.117	0.106

Each experiment was carried out based on the following procedure detailed in Section 4.2 and subsequently preprocessed according to the method described in Section 4.2.1.

**Fig. 5:**
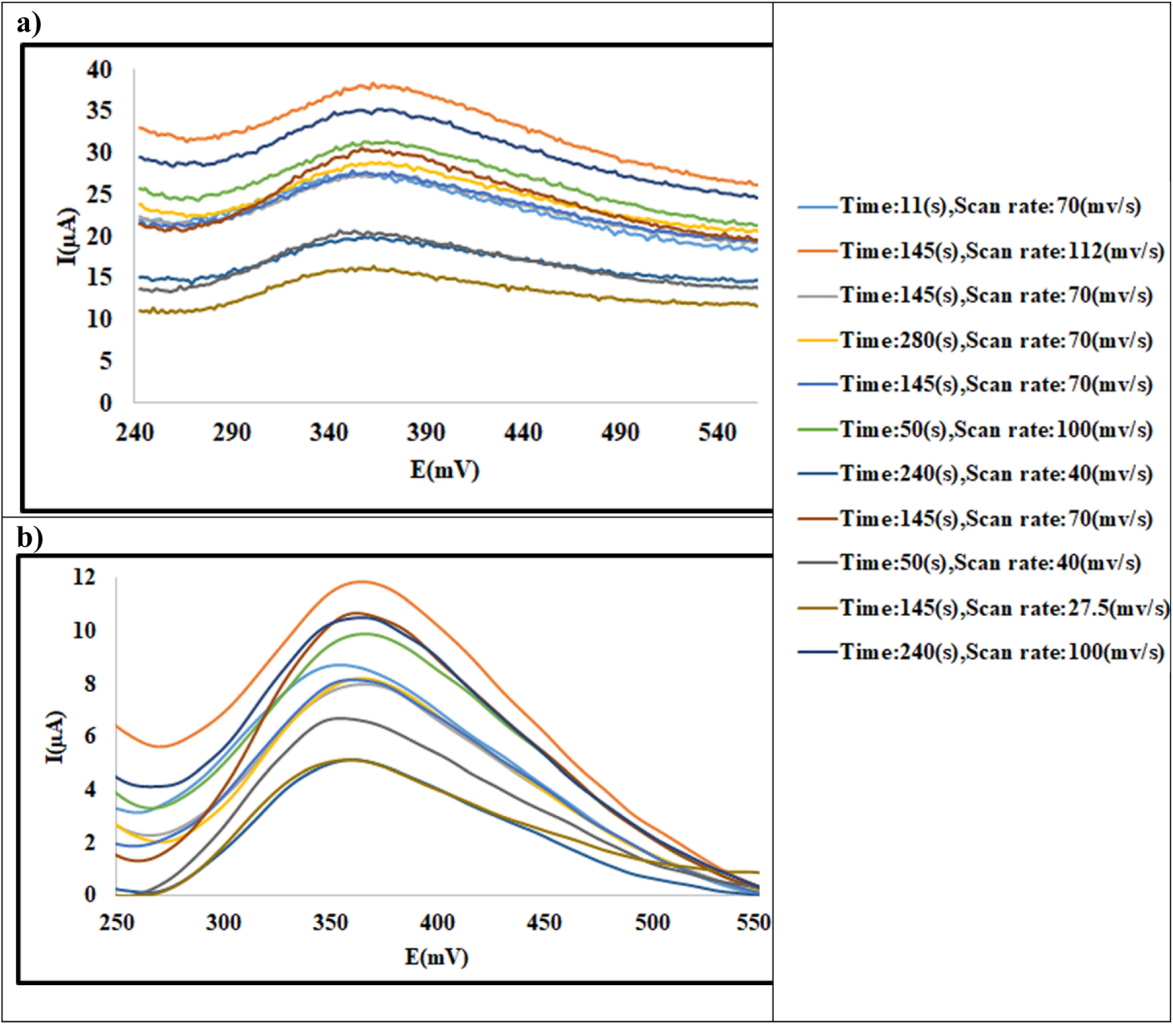
SWAS voltammograms of 2-NP (60 ppm) at the Ni-MOF74/Fe_3_O_4_/SiO_2_/NH_2_/β-CD/GCE surface in the PBS (0.1 M, pH = 9.40) at different scan rate (27.5–112 mv s^−1^) and different duration time (11–240 s), pulse = 500 mV. Experimental data (a) and pretreated data (b).

Fig. 5a illustrates the CCD data for 60 ppm of 2-NP and Fig. 5b shows its corresponding preprocessed data. For the other samples, the data were shown in SI (Fig. S2–S8). Then the anodic peak current was used as response at each experiment as reported in Table 1, where *Y*_2-NP10_, *Y*_2-NP30_, *Y*_2-NP60_ and *Y*_2-NP100_ denote as the anodic peak current for 10 ppm, 30 ppm,60 ppm and 100 ppm of 2-NP solutions, respectively; similarly, *Y*_4-NP10_, *Y*_4-NP30_, *Y*_4-NP60_ and *Y*_4-NP100_ denote as those of 4-NP, respectively.

Each CCD dataset was analyzed using RSM to establish the relationship between the responses and the parameters based on second order polynomial equation. The model quality was evaluated based on analysis of variance (ANOVA). Moreover, the most influential effects of factors and their interactions were examined based on ANOVA strategy.

Second order fitted models for all responses can be represented as follows:

7 *Y*_2-NP10_ = −0.15398 + 7.23195 × 10^−3^*A* − 3.52746 × 10^−5^*A*^2^

8 *Y*_4-NP10_ = +0.056545 + 9.74057 × 10^−4^*A*

9 *Y*_2-NP30_ = +0.049765 + 1.41291 × 10^−3^*A* − 2.68556 × 10^−4^*B* + 2.10351 × 10^−6^AB − 1.00579 × 10^−6^*A*^2^ + 4.47944 × 10^−7^*B*^2^

10 *Y*_4-NP30_ = +0.052478 − 1.12738 × 10^−4^*A* + 3.57695 × 10^−4^*B* −1.10351 × 10^−6^AB + 9.72836 × 10^−6^*A*^2^ − 1.02333 × 10^−6^*B*^2^

11 *Y*_2-NP60_ = +0.055240 + 1.66304 × 10^−3^*A* − 8.36719 × 10^−5^*B* + 3.24444 × 10^−6^AB − 6.25521 × 10^−6^*A*^2^ − 6.23404 × 10^−7^*B*^2^

12 *Y*_4-NP60_ = +0.029759 + 1.08410 × 10^−3^*A* + 4.98862 × 10^−5^*B* − 1.05265 × 10^−6^AB − 1.06207 × 10^−6^*A*^2^ − 8.71276 × 10^−8^*B*^2^

13 *Y*_2-NP100_ = −0.011038 + 2.27189 × 10^−3^*A* + 1.51809 × 10^−4^*B* − 2.59991 × 10^−6^AB − 5.93330 × 10^−6^*A*^2^ − 2.30687 × 10^−7^*B*^2^

14 *Y*_4-NP100_ = +0.085366 − 1.62043 × 10^−4^*A* − 3.63879 × 10^−4^*B* + 1.22193 × 10^−6^AB + 4.13447 × 10^−6^*A*^2^ + 7.89612 × 10^−7^*B*^2^

where *A*, *B*, *Y*_2-NP10_, *Y*_2-NP30_, *Y*_2-NP60_, *Y*_2-NP100_, *Y*_4-NP10_, *Y*_4-NP30_, *Y*_4-NP60_ and *Y*_4-NP100_ are the are the same parameters as mentioned above for Table 1; and AB, *A*^2^ and *B*^2^ represent the interaction effect of A and B, square effect of *A* and square effect of *B*, respectively.

Form eqn (7)–(14), it is deducted that the electrochemical behavior the electrode is severely dependent on the concentration of analyte, as observed in preliminary experiments.

A brief summary of the ANOVA results for *Y*_2-NP60_ is presented in Table 3, and the other responses reported in Tables S1–S7.

**Table 3:** Analysis of variance (ANOVA) result for the model of 2-NP60

Source	Sum of squares	d*f*	Mean square	*F*-value		*p*-Value prob > *F*
Model	0.012	5	2.419 × 10^−3^	8.88	0.0158	Significant
*A*–*A*	0.011	1	0.011	41.94	0.0013	
*B*–*B*	1.087 × 10^−4^	1	1.087 × 10^−4^	0.40	0.5554	
AB	3.420 × 10^−4^	1	3.420 × 10^−4^	1.26	0.3135	
*A* ^2^	1.767 × 10^−4^	1	1.767 × 10^−4^	0.65	0.4572	
*B* ^2^	1.796 × 10^−4^	1	1.796 × 10^−4^	0.66	0.4538	
Residual	1.362 × 10^−3^	5	2.725 × 10^−4^			
Lack of fit	1.367 × 10^−4^	3	4.558 × 10^−5^	0.074	0.9682	Not significant
Pure error	1.226 × 10^−3^	2	6.128 × 10^−4^			
Cor total	0.013	10				

The statistical evaluation of all models was conducted based on *p*-values. According to ANOVA strategy, the model is statistically significant when both of the following conditions are: the *p*-value of the model <0.05 and *p*-value of lack of fit (LOF) >0.05. Table 4 represents a summary of the ANOVA results and the regression coefficients of the samples. As shown in Table 3, the *p*-values for all models are less than 0.02, while the *p*-values of LOF are greater than 0.2, indicating that all the models are statistically adequate. Additionally, the regression coefficients including *R*^2^ and *R*_adj_^2^, were estimated to assess the goodness of the fit of experimental data to the quadratic model.

**Table 4:** Summery of analysis of variance (ANOVA) for central composite design

Analytes	*p*-Value	Lack of fit	Regression coefficient	
			*R* ^2^	*R*_adj_^2^
2-NP10	0.0008	0.8234	0.8322	0.7903
4-NP10	0.0148	0.9966	0.5010	0.4455
2-NP30	0.0028	0.2209	0.9505	0.9010
4-NP30	0.0240	0.8096	0.8794	0.7588
2-NP60	0.0158	0.9682	0.8988	0.7975
4-NP60	0.0137	0.8718	0.9047	0.8095
2-NP100	0.0011	0.8898	0.9663	0.9326
4-NP100	0.0277	0.6916	0.8718	0.7437

For all models, the relevant statistical parameters are reported in Table 4. In each model, the *R*^2^ and *R*_adj_^2^ values indicate a satisfactory correlation between the response and the independent factors. Indeed, the high values of these coefficients confirm that the applied models fit the experimental data well and accurately interpret the data.

**Fig. 6:**
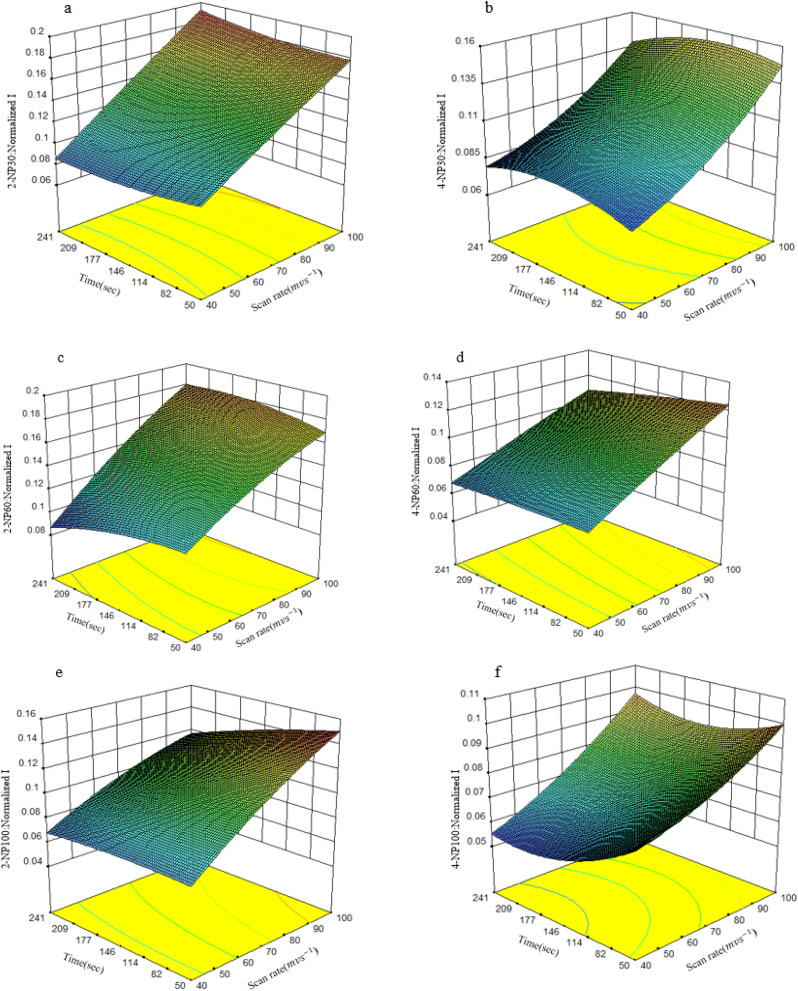
The 3-D response surface plots of 2-NP and 4-NP in buffer solution condition as a representative analyte, when two factors are fixed at center point and the others are variables.

To illustrate the effect of factor interactions on response, the response surface 3-D plots were used to visualize the relationship between the current, as response, and the experimental factors. Here, some 3-D plots for 2-NP and 4-NP have been shown in Fig. 6.

For all responses shown in Fig. 6, the curvature in the response *vs.* scan rate and accumulation time indicates an interaction between factors. Fig. 6a shows that for 30 ppm of 2-NP as scan rate and the duration time increase, the normalized current gently increases. This is because, at longer accumulation time, more analyte is deposited on the surface of the modified GCE electrode. Since the electrode surface is not saturated at this concentration, even at a high scan rate, sufficient time is available for the complete oxidation of the accumulated analyte. However, Fig. 6c illustrates that for 60 ppm of 2-NP, the simultaneous increasing of two factors leads to increasing the current and then results in a decrease thereafter. It can be noted that as the accumulation time increases, a greater amount of analyte accumulates at 60 ppm of 2-NP. Since the electrode requires more time to oxidize the analyte, a slower oxidation process occurs at high scan rates, consequently, the anodic peak currents of 2-NP decrease. Fig. 6e shows the same observation as that of Fig. 6b, while the decrease in signal at high scan rates and duration time is more outstanding than that of Fig. 6b due to the high concentration of 2-NP (100 ppm). Fig. 6b, d and f for 4-NP at different concentration show the similar trend in dependency of signals to the levels of factors as discussed for 2-NP.

#### 4.2.3. Multiple response surface methodology to find the optimal conditions of nitrophenol oxidation at Ni-MOF74/Fe_3_O_4_/SiO_2_/NH_2_/β-CD/GCE

Desirability function is the most commonly used strategy in RSM to optimize processes involving multiple responses simultaneously.^31^ This method combines several responses into a single accumulated function which is used for optimization purpose. Each predicted response is transformed into a desirability value (*df*_*i*_) with a range of zero (completely undesirable response) to one (fully desirable response) according to the Derringer and Suich equation as follows:^31^

15 *df*_*i*_ = (*R* − *α*/*β* − *α*)^*w*_*i*_^, *α* ≤ *R* ≤ *β*

16 *df*_*i*_ = 1*R* > *β*

17 *df*_*i*_ = 0*R* < *α*

Here, *α* and *β* represent the low and high extremes of each response, respectively, and *w*_*i*_ denotes the importance of each response. The overall optimization function (DF) is computed as the geometric mean of individually *df*_*i*_, as shown below:18
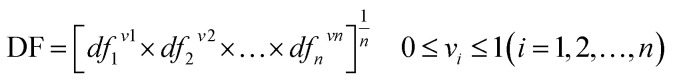
19
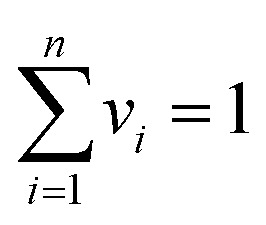
In this equation, *df*_*i*_ denotes the individual desirability of *i*th response *R*_*i*_ (*i* = 1, 2, 3, …, *n*) and *v*_*i*_ is corresponding weight assigned to that response. The overall DF reflects the combined performance of all responses which can be optimized within the defined range of independent variables domain using univariate search methods.

**Fig. 7:**
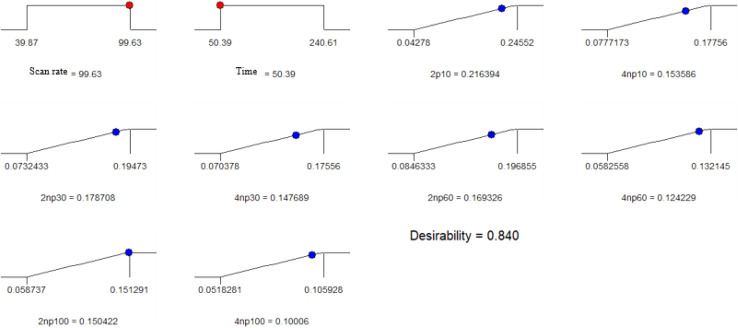
3-D desirability plot *vs.* scan rate and duration time to optimize 2-NP and 4-NP oxidation process.

The aim of this study is to identify common optimal conditions for the oxidation processes of 2-NP and 4-NP where the maximum anodic peak currents for both analytes are achieved across the entire concentration range of calibration curve. The optimal condition was obtained based on maximizing all responses (eqn (15)–(19)), the desirability plot as a function of the factors was shown in SI (Fig. S9). The results revealed the following condition as the optimal one (Fig. 7): 99.63 mv s^−1^ of scan rate and 50.39 s of duration time. This condition was used to quantify the analytes in their mixtures.

## 5. Simultaneous quantification of 2-NP and 4-NP in binary solutions

**Fig. 8:**
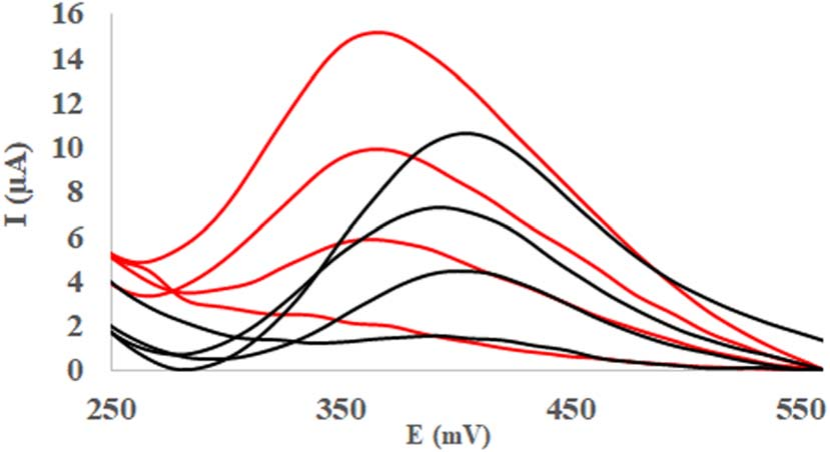
Desirability plot of 2-NP, 4-NP in buffer solution condition.

Under the obtained above optimized condition, first the analytical performance of the modified-GCE was investigated for individual analytes at the concentration range of (10–100 ppm). Fig. 8 shows the voltammograms of each analyte at different concentration and Table 5 reports the results of the univariate calibration of each analyte, plotting the anodic peak current *vs.* concentration. As seen from Table 5, the calibration curve of each analyte is linear with high *R*^2^ value for both analytes.

**Table 5:** Calibration parameters for univariate calibration of analytes at the optimal condition

Statistical parameters	2-NP	4-NP
Slope	0.143 (±0.008)	0.099 (±0.008)
*R* ^2^	0.9942	0.9859
RMSE	0.5	0.6

The main purpose of this work is simultaneous determination of 2-NP and 4-NP in their binary mixtures while it can be observed in Fig. 8 the signals are severely overlapped. As discussed above, in this range of calibration, both signals of analytes are linearly correlated with their concentrations. Therefore, simultaneous quantification of the analytes can be conducted based on linear multivariate calibration techniques such as PCR and PLS. In this purpose, a sample set containing different concentrations of 2-NP and 4-NP were synthesized based on 4-level full factorial design (Table 6) and these samples were augmented with those of used in calibration. The sample set consists of 24 sample whose corresponding data are illustrated in Fig. S10.

**Table 6:** Different concentrations of 2-NP and 4-NP, based on 4-level full factorial design

Sample no.	2-NP (ppm)	4-NP (ppm)
1	10	0
2	30	0
3	60	0
4	100	0
5	0	10
6	0	30
7	0	60
8	0	100
9	10	10
10	10	30
11	10	60
12	10	100
13	30	10
14	30	30
15	30	60
16	30	100
17	60	10
18	60	30
19	60	60
20	60	100
21	100	10
22	100	30
23	100	60
24	100	100

**Fig. 9:**
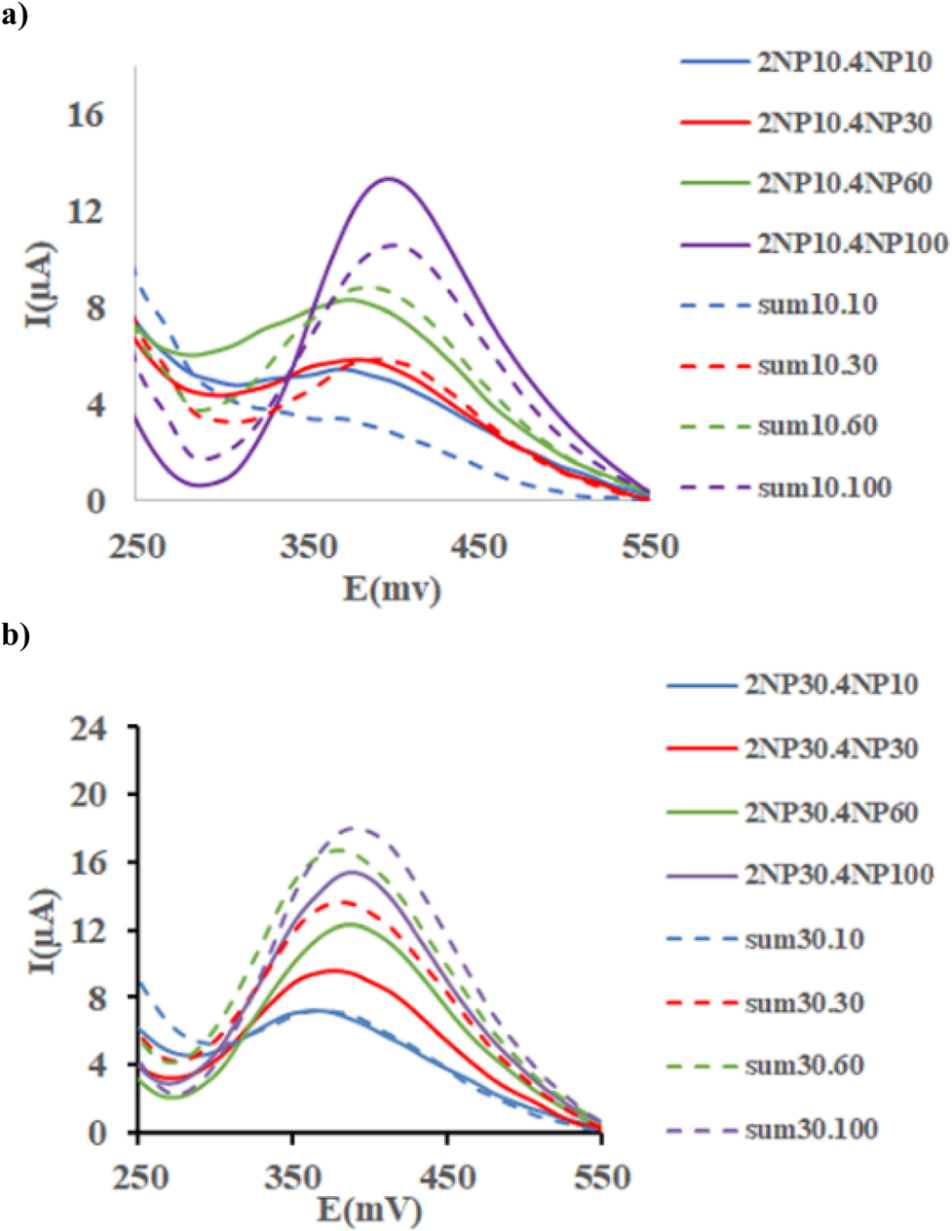
(a) SWAS voltammograms of 2-NP (10 ppm) with 2-NP (10–100ppm); and (b) 2-NP (30 ppm) with 4-NP (10–100ppm) at the Ni-MOF74/Fe_3_O_4_/SiO_2_/NH_2_/β-CD/GCE surface in the PBS (0.1 M, pH = 9.40) at scan rate 100 and duration time 50 s, pulse = 500 mV.

**Fig. 10:**
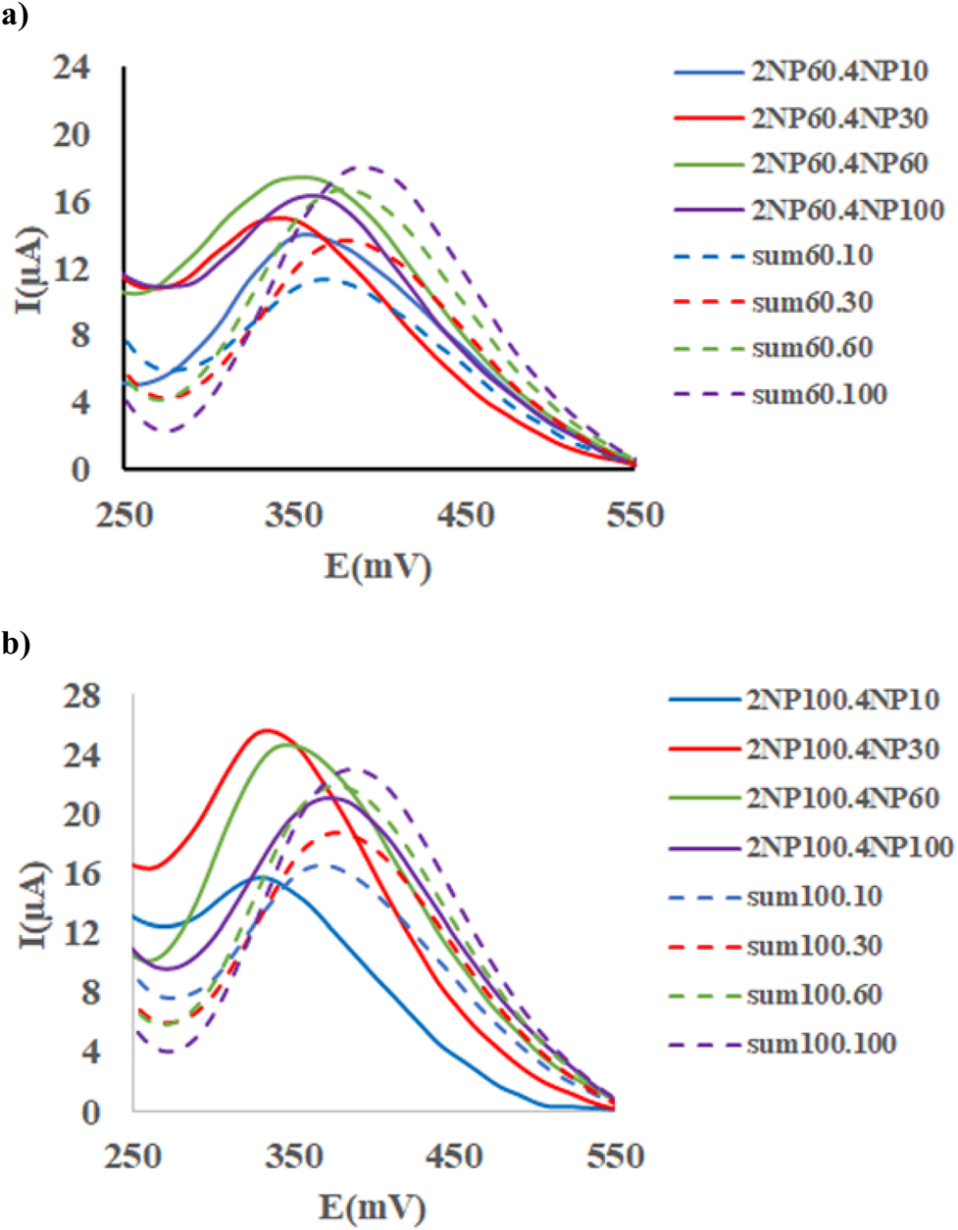
(a) SWAS voltammograms of 2-NP (60 ppm) with 2-NP (10–100ppm); and (b) 2-NP (1000 ppm) with 4-NP (10–100ppm) at the Ni-MOF74/Fe_3_O_4_/SiO_2_/NH_2_/β-CD/GCE surface in the PBS (0.1 M, pH = 9.40) at scan rate 100 and duration time 50 s, pulse = 500 mV.

As much as, the matrix effects are common problem in the electrochemical analysis, this problem was examined by superimposing the voltammograms of 10 ppm of 2-NP with those obtained from successive standard additions of 4-NP (Fig. 9a). Moreover, for each sample, the voltammograms of the corresponded standard solution of the analytes were combined and the resulting sum was superimposed in Fig. 9a. As seen, clearly observed, the summed individual signals of analytes differ significantly from their corresponding signals in the mixture, indicating the strong matrix effect. Moreover, this effect appears to depend on the initial concentration of 2-NP, as seen in Fig. 9b, 10a and b, where successive addition of 4-NP was performed to solutions containing 30, 60 and 100 ppm of 2-NP, respectively. The same visualization was conducted for 4-NP analyte where different concentration of 2-NP were spiked to 10, 30, 60 and 100 ppm of 4-NP as shown in SI (Fig. S11 and S12).

First, the data was analyzed by PLS as a linear model, however, the results showed that PLS model did not lead to accurate and reliable results as *R*^2^ value was too low to be considered acceptable. From the above detailed analysis, it was deduced that there are three main problems in simultaneous determination 2-NP and 4-NP based on electrode modified with Ni-MOF74/Fe_3_O_4_/SiO_2_/NH_2_/β-CD, as follows: sever signal overlapping, drastic matrix effect and non-linearity, a fact which made us to apply non-linear artificial neural network method for analysis of this data set.

## 6. Optimization of the neural network

The concentrations of 2-NP and 4-NP in the sample set (Table 6) were first coded according to eqn (5). The dataset was then randomly divided into training (18 samples, 75%), validation (3 samples, 12.5%), and test (3 samples, 12.5%) subsets. The training set was used to adjust network weights, while the validation set served to prevent overfitting and determine the optimal number of neurons in the hidden layer. The test set was held out entirely from training and used exclusively for final performance evaluation, ensuring that reported prediction metrics reflect the network's ability to predict unseen data.

The anodic peak currents of 2-NP and 4-NP were employed as input neurons. Inputs were encoded and divided into the aforementioned subsets. Training was conducted with random initialization of weights and repeated multiple times to minimize dependency on initial conditions. Two statistical parameters—*R*^2^ and mean squared error (MSE)—were used to assess network performance and guide the selection of the optimal hidden layer topology (eqn (20)). The following equation represents the MSE parameter:20
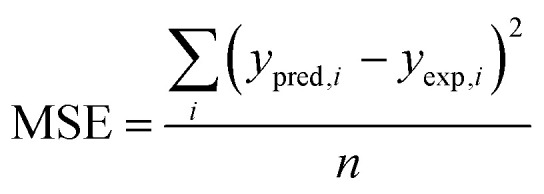
where *y*_pred,*i*_ and *y*_exp,*i*_ are the predicted value of 2-NP concentration from ANN in the ith sample of training set and its corresponding real concentration, respectively, and *y*_m_ is the average of concentrations and *n* is the number of samples in the training set. The same parameters can be computed for two other sets.

**Fig. 11:**
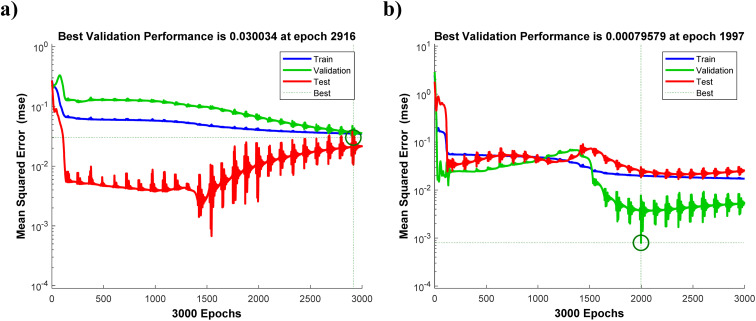
Mean square error *vs.* number of epochs in ANN optimization for (a) 2-NP and (b) 4-NP.

For each analyte, the number of hidden neurons and training epochs were optimized based on MSE trends across training and validation sets (Fig. 11). As illustrated in Fig. 11, the minimum MSE was obtained at two hidden neurons with 2916 number of epochs for 2-NP, and three hidden neurons with 1997 number of epochs for 4-NP, ensuring minimal error and prevention of overfitting. Table S8 summarizes key ANN parameters, including network topology, number of data points in each subset, and training algorithm. Fig. S13 illustrates the network architectures, showing input, hidden, and output neurons for both analytes.

The goodness-of-fit of the ANN model was evaluated using *R*^2^ and root mean square error (RMSE) for all datasets (eqn (21)), with results presented in Table 7. RMSE is writtens as follows:21
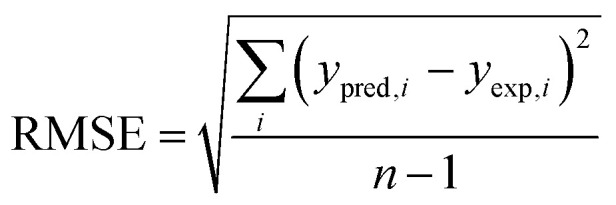
Here, *y*_pred,*i*_ and *y*_exp,*i*_ and *n* are the same parameters as introduced in eqn (20).

**Table 7:** Statistical parameters of the ANN model

Data set	2-NP		4-NP	
	*R* ^2^	RMSE	*R*-square	RMSE
Total	0.9303	8.2	0.9604	8.9
Training	0.9430	8.4	0.9382	10.3
Test	0.8889	9.5	0.9857	8.5
Validation	0.9113	5.9	0.9914	1.5

**Fig. 12:**
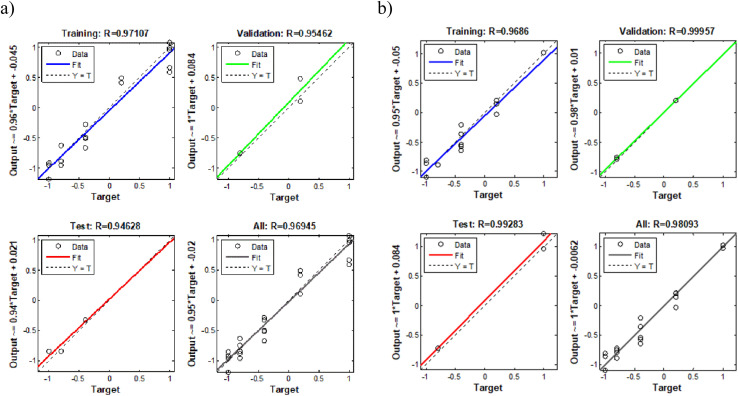
Comparison between experimental and predicted results for different fitted data sets: (a) 2-NP and (b) 4-NP.

Fig. 12 compares experimental and predicted concentrations, confirming the network's capability to accurately quantify analytes with strongly overlapping signals and non-linear matrix effects, demonstrating reliable performance for complex sample matrices.

## 7. Effect of input variables

The ANN's weights are a series of numerical values that make connection between the input currents and the hidden layer neurons, as well as between the higgen neurons and concentration of analyte in out layer. The neural weight matrix, which can be used to assess the relative importance of each current on the output variable, was determined using numerical approach proposed by the Garson method, as follows:^54^22
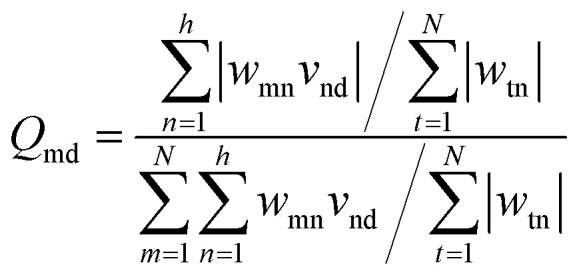
Here, *w*_tn_ represents the weight between the *m*_th_ input neuron and the *n*_th_ hidden neuron, while *v*_nd_ denotes the weight between the *n*_th_ hidden neuron and the *d*_th_ output neuron. For each analyze, the final ANN model estimated the percentage influence of input variables on analyte concentration by combining the weights of both input-hidden and hidden-output connections. Table 8 presents the results, revealing that for 2-NP, the first input has greater than importance than the second one. Interestingly, this order implies that the peak current of 2-NP is more significant than that of 4-NP. Conversely, for 4-NP analysis, the opposite order is observed, as reported in Table 9. These results highlights the effectiveness of ANN modelling in analyzing highly complex and non-linaer data presented in this work.

**Table 8:** Effective weight matrix for 2-NP analysis

Input currents		Hidden neurons	Hidden to out
2-NP peak current	4-NP peak current		
−1.511	2.358	H1	−0.271
2.728	−0.632	H2	0.329
−2.119	1.830	H3	−0.370
0.931	2.641	H4	0.771
59	41	Relative importance %	

**Table 9:** Effective weight matrix for 4-NP analysis

Input currents		Hidden neurons	Hidden to out
2-NP peak current	4-NP peak current		
−1.657	1.770	H1	−0.480
−0.402	−2.391	H2	0.756
−1.498	1.907	H3	−0.623
0.36	0.64	Relative importance %	

## 8. Comparison with other work

To the best of our knowledge, only a limited number of studies have reported the simultaneous determination of nitrophenols using electrochemical sensors, as summarized in Table 8.

Based on Table 10, conventional electroanalytical techniques suffer from several drawbacks, including the toxicity associated with mercury electrodes in differential pulse voltammetry (DPV)^55,56^ and the time-consuming nature of conductometric titrations.^58^ Even bismuth-modified pencil-lead electrodes,^57^ while more environmentally friendly than mercury-based systems, still present limitations such as lower surface area, weaker sensitivity, and reduced long-term stability compared with advanced the prepared MOF-modified electrode. In contrast, the proposed SWASV with a MOF-modified electrode provides a mercury-free and eco-friendly alternative, offering enhanced electrode stability and faster analysis.

**Table 10:** Comparison of different electrochemical methods that were used for simultaneous quantification of some nitrophenols

Method	Electrode type	LDR (ppm)	References
Differential pulse polarography-SVM	Dropping-mercury electrode (DME)	0.097–97.37(2-NP)	55
		0.069–69.55(4-NP)	
Differential pulse voltammetry-PLS, PCR, CLS	Hanging mercury drop electrode (HMDE)	0.1–2 (2-NP)	56
		0.1–2 (4-NP)	
Differential pulse voltammetry-NASSAM	Modified pencil-lead electrode with bismuth	0.278–27.8 (2-NP)	57
		0.556–55.6 (4-NP)	
Conductometry acid–base titration-ANN	—	0.6–1.9 (4-NP)	58
Square wave anodic stripping voltammetry-ANN	Ni-MOF-74/Fe_3_O_4_/SiO_2_/NH_2_/β-CD/GCE	1–100	Our study

Compared with linear modeling approaches such as partial least squares (PLS), principal component regression (PCR), and classical least squares (CLS),^56^ or combined chemometric strategies such as the net analyte signal–standard addition method (NAS–SAM),^57^ our method achieves a wider linear dynamic range (LDR) and superior ability to capture non-linear features in electrochemical data. In particular, even compared with methods integrated with ANN,^55,58^ our approach still provides a broader dynamic range. This improvement arises from the use of MOFs to modify the electrode surface, where the high surface area, porous architecture, and tunable chemical functionality significantly increase the number of active sites and facilitate stronger current responses. Moreover, the structural stability and compositional tunability of MOFs contribute to improved robustness, reproducibility, and long-term operational stability. These features highlight the superior performance of MOF-modified electrodes over previously reported electrodes, including those based on bismuth–graphite materials.

Considering that the maximum permissible concentration of nitrophenols in environmental samples is approximately 20 ppm, the proposed strategy offers sufficient sensitivity and an appropriate linear range to ensure reliable detection and quantification in real matrices. Overall, coupling the MOF-based electrochemical sensor with ANN provides an effective strategy to address signal overlap and non-linear responses in complex electrochemical systems. This integrated approach enhances data interpretation and predictive accuracy, ultimately offering a powerful tool for reliable environmental monitoring of nitrophenols.

## 9. Conclusions

The quantification of nitrophenols (NPs) is critically important for assessing environmental pollution. Among various detection techniques, electrochemical sensors offer remarkable advantages such as cost-effectiveness, rapid response, ease of miniaturization, simple operation, and the capability for *in situ* detection. In this study, we highlight the potential of combining advanced materials with machine learning for the simultaneous determination of 2-NP and 4-NP in aqueous solutions. A novel nanostructured electrochemical sensor was developed based on a nickel–organic framework (Ni-MOF74), Fe_3_O_4_/SiO_2_/NH_2_ nanoparticles, and β-cyclodextrin (β-CD), all incorporated into a SiO_2_ matrix. This composite was employed as a voltammetric sensor for analyte quantification using the SWASV method.

The electrochemical data were highly complex due to signal overlap between the analytes and strong non-linear matrix effects, which were effectively addressed by employing an ANN for data analysis. The calibration results yielded *R*^2^ values of 0.93024 and 0.9604 for 2-NP and 4-NP, respectively, confirming the capability of the ANN to achieve accurate simultaneous electrochemical quantification of nitrophenols in aqueous solutions. A comparison with previously reported studies revealed that our method provides a significantly broader linear dynamic range while maintaining sufficient detection limits for environmental applications, including the analysis of contaminated samples, industrial effluents, and situations involving higher concentration levels.

While the developed Ni-MOF74/Fe_3_O_4_/SiO_2_/NH_2_/β-CD/GCE sensor coupled with ANN modeling demonstrated excellent performance for simultaneous quantification of 2-NP and 4-NP, two main directions remain for future improvement. First, integration of the electrochemical sensor into a portable, field-deployable device would enable real-time, on-site environmental monitoring. Second, retraining and validating the ANN model using real environmental samples—containing potential interferents—would further enhance the robustness and practical applicability of the proposed method.

## Conflicts of interest

There are no conflicts to declare.

## Supplementary Material

RA-015-D5RA03838C-s001

RA-015-D5RA03838C-s002

RA-015-D5RA03838C-s003

## Data Availability

The data supporting this article have been included as part of the supplementary information (SI). Supplementary information: experimental data are available as excel file; some figures and tables were reported in word files. See DOI: https://doi.org/10.1039/d5ra03838c.

## References

[cit1] Megharaj M., Pearson H. W., Venkateswarlu K. (1991). Arch. Environ. Contam. Toxicol..

[cit2] Keith L., Telliard W. (1979). Environ. Sci. Technol..

[cit3] Tang Q., Huang L., Chen T., Xie F., Tan H., Chen D. (2025). J. Fluoresc..

[cit4] Narayanan S., Sakthivel P., Venkataraman B. (2025). Electrocatalysis.

[cit5] Boddu V. (2017). et al.. Int. J. Environ. Anal. Chem..

[cit6] Wang S., Li Y., Song J., Zhang J., Ma Y. (2023). J. Electroanal. Chem..

[cit7] Al-Shaalan N. H. (2021). et al.. Sep. Sci. Technol..

[cit8] Wang S. (2023). et al.. Microchem. J..

[cit9] Naik S. S. (2021). et al.. J. Hazard. Mater..

[cit10] Veerapandi G., Govindan R., Sekar C. (2023). Chemosphere.

[cit11] Li J. (2020). et al. Electrochim. Acta.

[cit12] Burgot J.-L. , General Analytical Chemistry: Electrochemical Analysis Methods, CRC Press, Boca Raton, 2024

[cit13] Nodehi M., Baghayeri M., Veisi H. (2021). Talanta.

[cit14] Ye M.-H. (2022). et al.. Electrochim. Acta.

[cit15] Scandurra A., Mirabella S. (2021). IEEE Sens. J..

[cit16] Baghayeri M. (2021). et al.. Mater. Chem. Phys..

[cit17] Liu L. (2018). et al.. ChemElectroChe.

[cit18] Gholami F. (2024). et al.. Microchem. J..

[cit19] Bhagawati P. B. (2024). et al.. Desalin. Water Treat..

[cit20] Keithley R. B., Wightman R. M. (2011). ACS Chem. Neurosci..

[cit21] Chang J., Song D. (2023). J. Food Meas. Char..

[cit22] Despagne F., Massart D. L. (1998). Analyst.

[cit23] Ma Y. , and Guo G., Support Vector Machines Applications, Springer, vol. 649, 2014

[cit24] Miljković D. , Electronics and Microelectronics (MIPRO), IEEE, 2017

[cit25] Liu J. , Radial Basis Function (RBF) Neural Network Control for Mechanical Systems: Design, Analysis and Matlab Simulation, Springer Science & Business Media, New York, 2013

[cit26] Lewis P. A., Stevens J. G. (1991). J. Am. Stat. Assoc..

[cit27] Peng X. (2019). et al.. Electroanalysis.

[cit28] Baghayeri M. (2018). et al.. J. Electroanal. Chem..

[cit29] Zhang X. (2011). et al.. J. Colloid Interface Sci..

[cit30] Hu Q. , Zhang M. and Peng J., et al., Research Square, 2023, preprint, pp. 1–16, 10.21203/rs.3.rs-3431188/v1

[cit31] Brown S. , Tauler R., and Walczak B., Comprehensive Chemometrics: Chemical and Biochemical Data Analysis, Elsevier, 2020

[cit32] Sajjadi S. M. (2021). et al.. New J. Chem..

[cit33] Niu C. (2022). et al.. Water Res..

[cit34] Jeong N., Chung T.-h., Tong T. (2021). Environ. Sci. Technol..

[cit35] Jawad J., Hawari A. H., Zaidi S. J. (2021). Chem. Eng. J..

[cit36] Çerçi K. N., Hürdoğan E. (2020). Int. Commun. Heat Mass Transfer.

[cit37] Zhang C.-N. (2020). et al.. J. Inorg. Organomet. Polym. Mater..

[cit38] Wang N. (2015). et al.. Chem. Eng. Sci..

[cit39] Amini S. (2020). et al.. Microchim. Acta.

[cit40] Gharagozlou M. (2012). J. Chin. Chem. Soc..

[cit41] Jahanbakhsh Z., Hosseinzadeh H., Masoumi B. (2021). J. Sol-Gel Sci. Technol..

[cit42] Tayebee R. (2017). et al.. Int. J. Nano Dimens..

[cit43] Xu R. (2020). et al.. Ionics.

[cit44] Adpakpang K. (2014). et al.. Electrochim. Acta.

[cit45] Dastneshan A. (2023). et al.. Chem. Eng. J..

[cit46] Abarca R. L. (2016). et al.. Food Chem..

[cit47] Shown I. , et al. in Macromolecular Symposia, Wiley Online Library, 2010

[cit48] Lopa N. S. (2018). et al.. J. Electroanal. Chem..

[cit49] Jia R. (2024). et al.. RSC Appl. Interfaces.

[cit50] Janani M., Senejani M. A., Isfahani T. M. (2021). Appl. Organomet. Chem..

[cit51] Zimmermann B., Kohler A. (2013). Appl. Spectrosc..

[cit52] Žunić M. J. (2014). et al.. Appl. Surf. Sci..

[cit53] Canizares P. (2004). et al.. Ind. Eng. Chem. Res..

[cit54] Garson G. D. , Hierarchical Linear Modeling: Guide and Applications, Sage, 2013

[cit55] Asadpour-Zeynali K., Soheili-Azad P. (2012). Environ. Monit. Assess..

[cit56] Ni Y., Wang L., Kokot S. (2001). Anal. Chim. Acta.

[cit57] Asadpour-Zeynali K., Najafi-Marandi P. (2011). Electroanalysis.

[cit58] Rounaghi G., Mohammad Zadeh Kakhki R., Heidari T. (2011). Ind. Eng. Chem. Res..

