# Supplementary material for: A novel MOF-based electrochemical sensor for simultaneous quantification of nitrophenols in binary mixtures using artificial neural networks: challenges and opportunities
Source: RSC Adv. 2025 Oct 29;15(49):41400–17. doi: 10.1039/d5ra03838c (PMC12570034; doi:10.1039/d5ra03838c)
Supplement: RA-015-D5RA03838C-s003 [file RA-015-D5RA03838C-s003.pdf]

Supplementary Information (SI) for RSC Advances.
This journal is © The Royal Society of Chemistry 2025

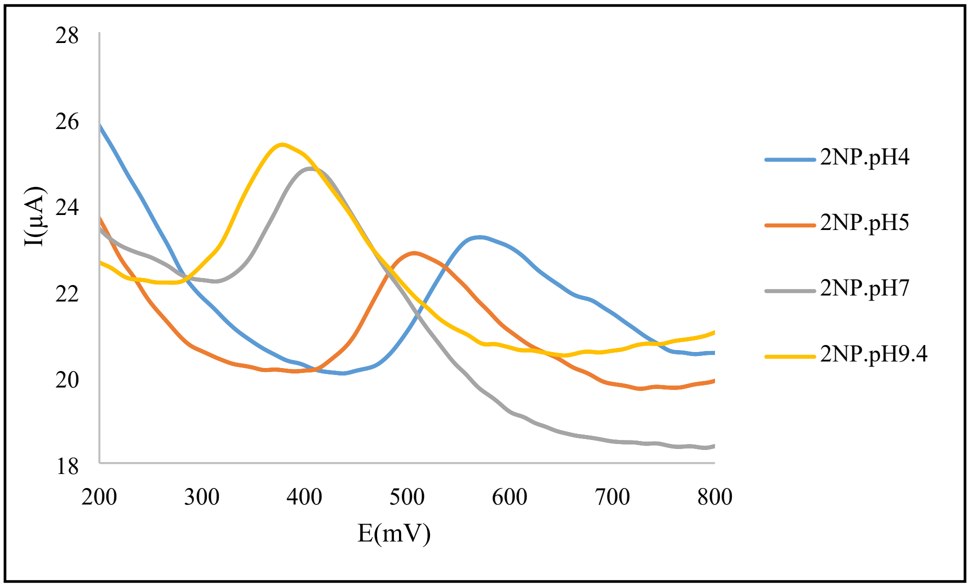

Fig. S1. SWAS voltammograms of 2-NP (30ppm) at the Ni-MOF74/ Fe_3O_4/SiO_2/NH_2/ β-CD / GCE surface in the PBS (0.1M) at different pH (4.00-9.40), scan rate 100 mvs^{-1},duration time120 s , Pulse=500 mV.

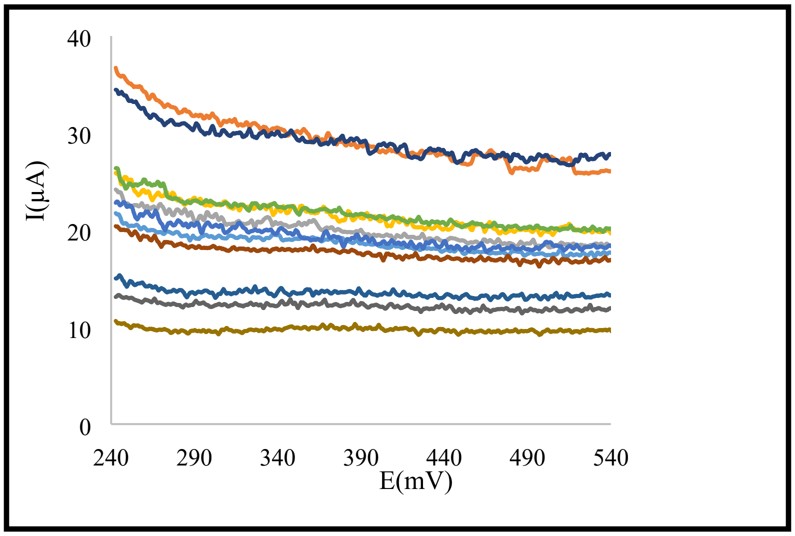

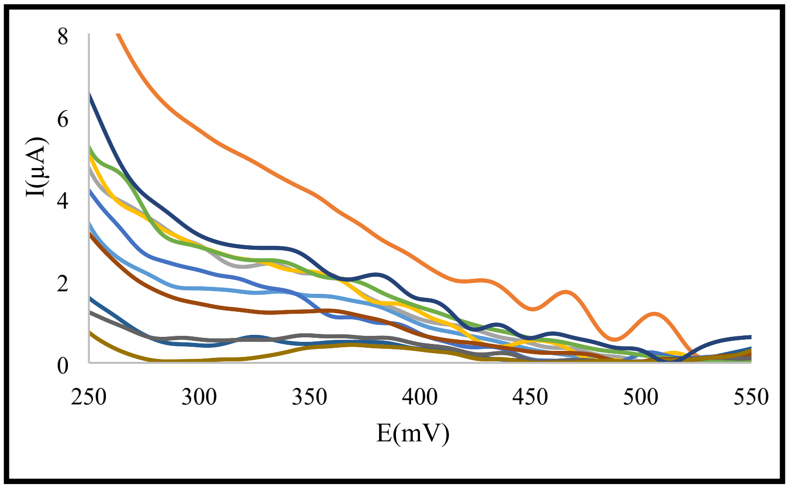

Fig. S2. SWAS voltammograms of 2-NP (10ppm) at the Ni-MOF74/ $Fe_3O_4/SiO_2/NH_2$/ β-CD / GCE surface in the PBS (0.1M, pH= 9.40) at different scan rate (27.5-112mvs^{-1}) and different duration time (11-240 s), Pulse=500 mV. Experimental data (a) and Pretreated Data (b)

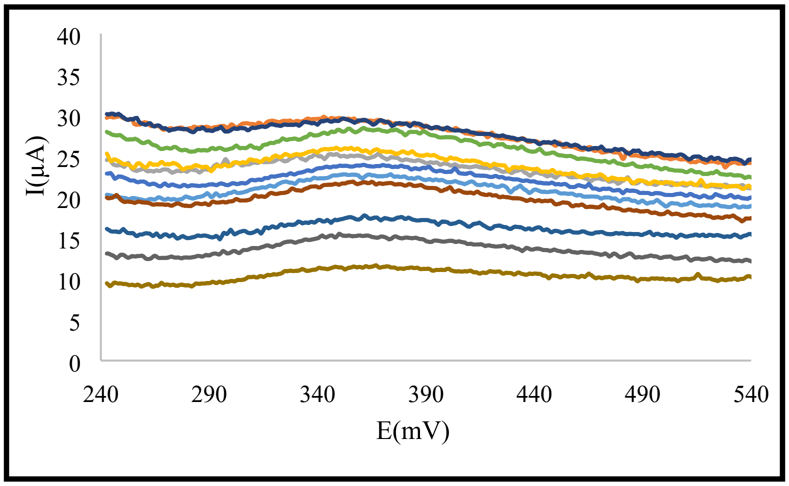

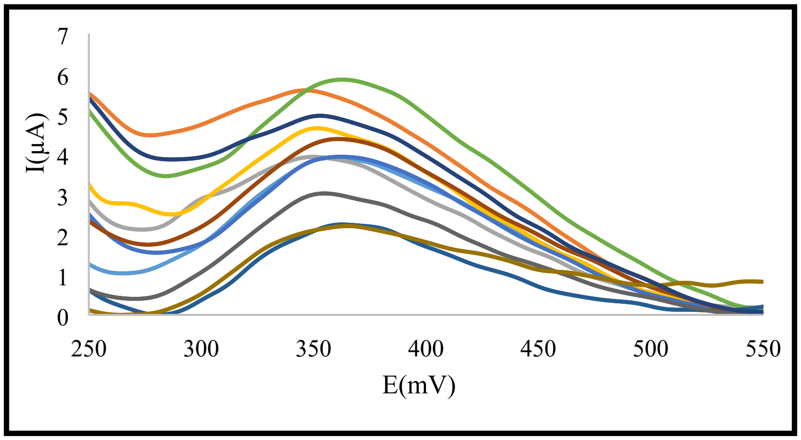

Fig. S3. SWAS voltammograms of 2-NP (30ppm) at the Ni-MOF74/ $Fe_3O_4/SiO_2/NH_2$/ β-CD / GCE surface in the PBS (0.1M, pH= 9.40) at different scan rate (27.5-112mvs^{-1}) and different duration time (11-240 s), Pulse=500 mV. Experimental data (a) and Pretreated Data (b)

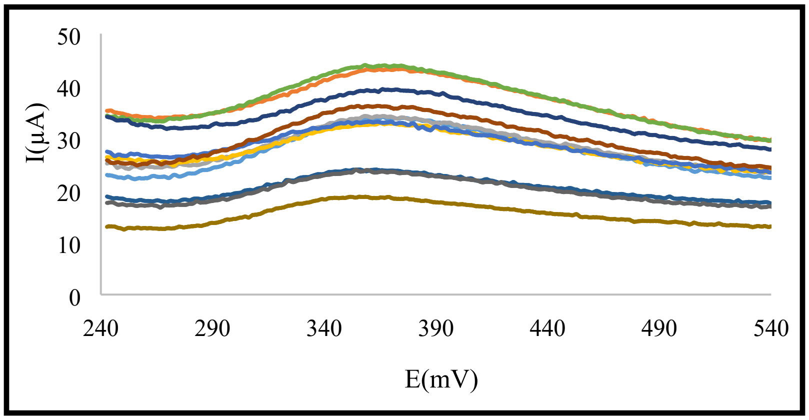

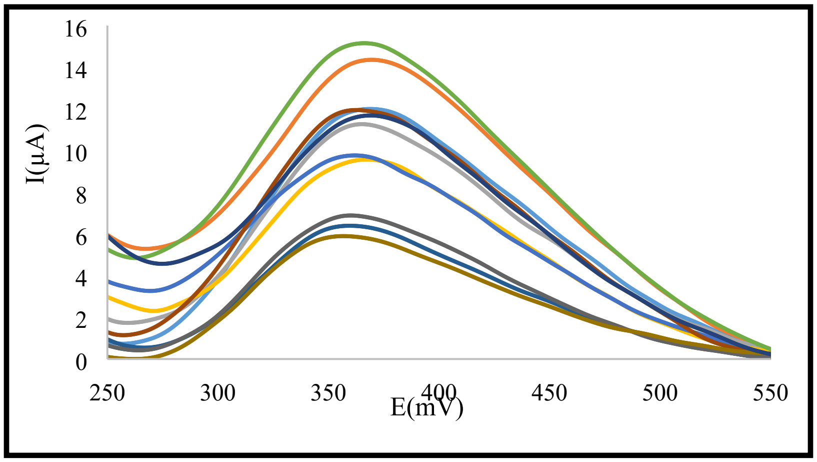

Fig. S4. SWAS voltammograms of 2-NP (100ppm) at the Ni-MOF74/ Fe_3O_4/SiO_2/NH_2/ β-CD / GCE surface in the PBS (0.1M, pH= 9.40) at different scan rate (27.5-112mvs^{-1}) and different duration time (11-240 s), Pulse=500 mV. Experimental data (a) and Pretreated Data (b)

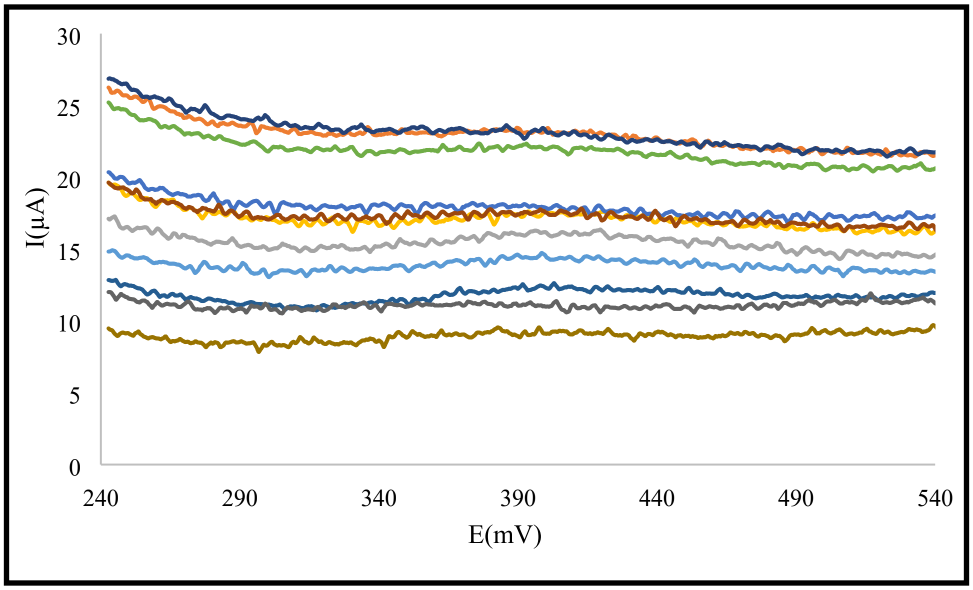

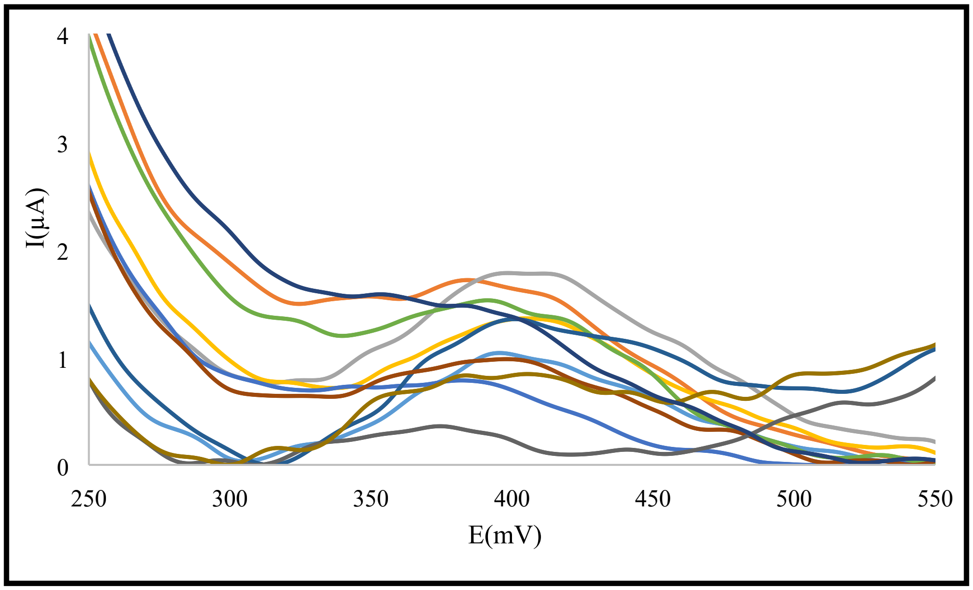

Fig. S5. SWAS voltammograms of 4-NP (10ppm) at the Ni-MOF74/ Fe_3O_4/SiO_2/NH_2/ β-CD / GCE surface in the PBS (0.1M, pH= 9.40) at different scan rate (27.5-112mvs^{-1}) and different duration time (11-240 s), Pulse=500 mV. Experimental data (a) and Pretreated Data (b)

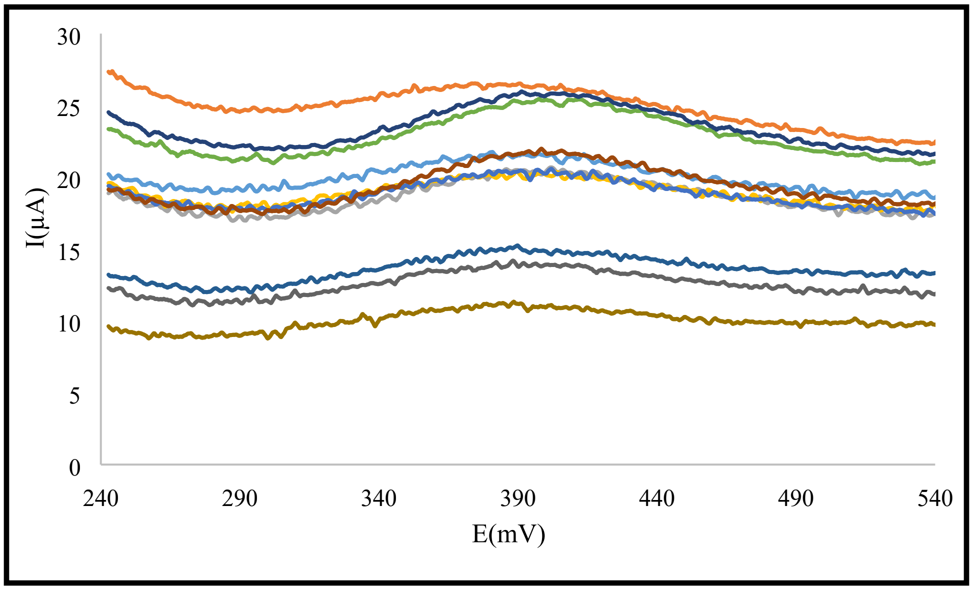

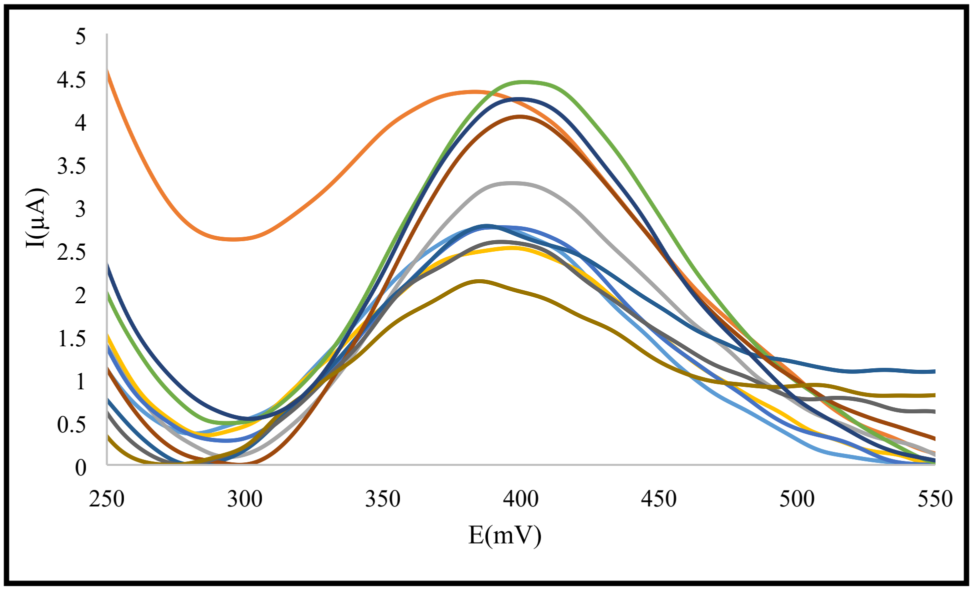

Fig. S6. SWAS voltammograms of 4-NP (30ppm) at the Ni-MOF74/ Fe_3O_4/SiO_2/NH_2/ β-CD / GCE surface in the PBS (0.1M, pH= 9.40) at different scan rate (27.5-112mvs^{-1}) and different duration time (11-240 s), Pulse=500 mV. Experimental data (a) and Pretreated Data (b)

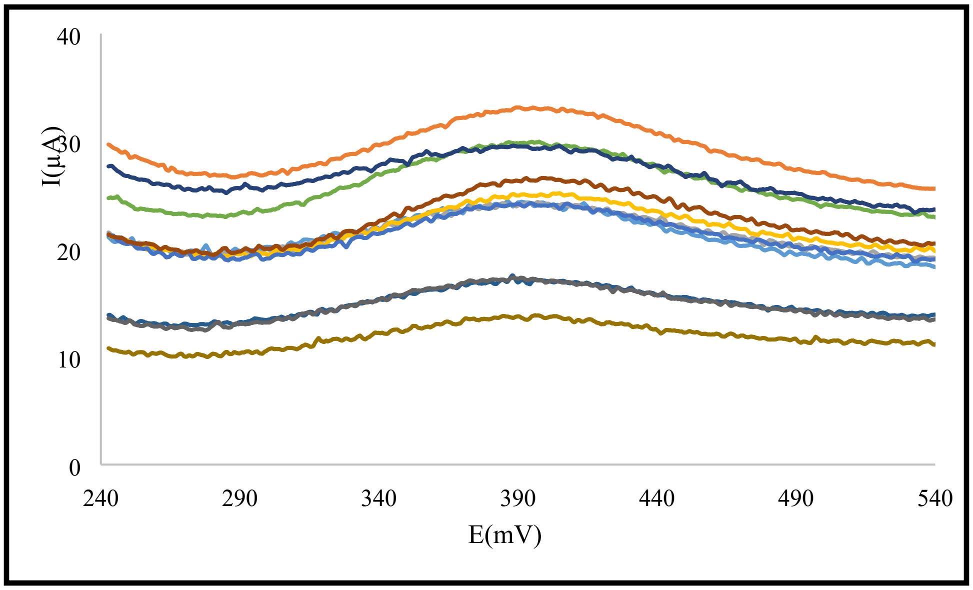

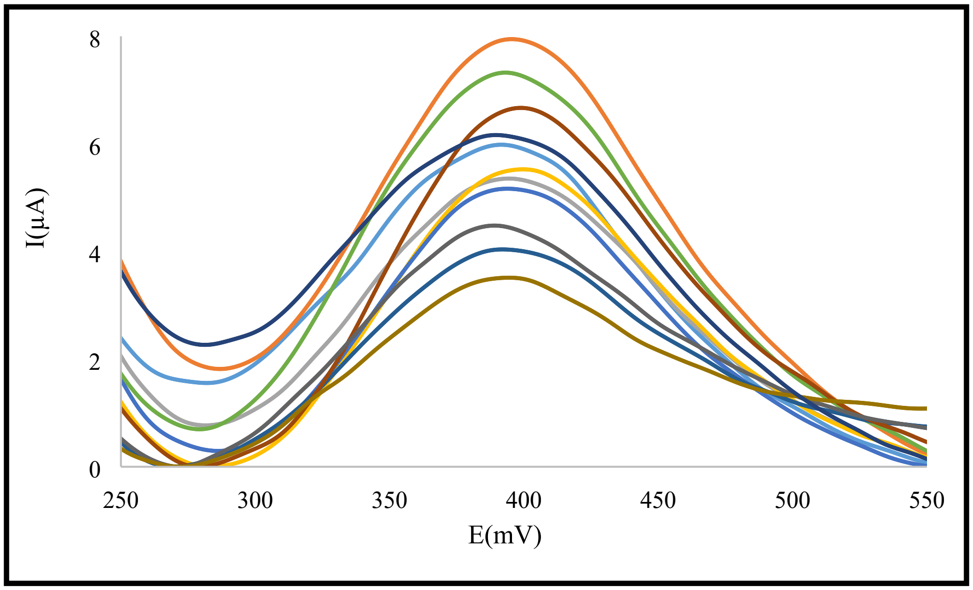

Fig. S7. SWAS voltammograms of 4-NP (60ppm) at the Ni-MOF74/ $Fe_3O_4/SiO_2/NH_2$/ β-CD / GCE surface in the PBS (0.1M, pH= 9.40) at different scan rate (27.5-112mvs^{-1}) and different duration time (11-240 s), Pulse=500 mV. Experimental data (a) and Pretreated Data (b)

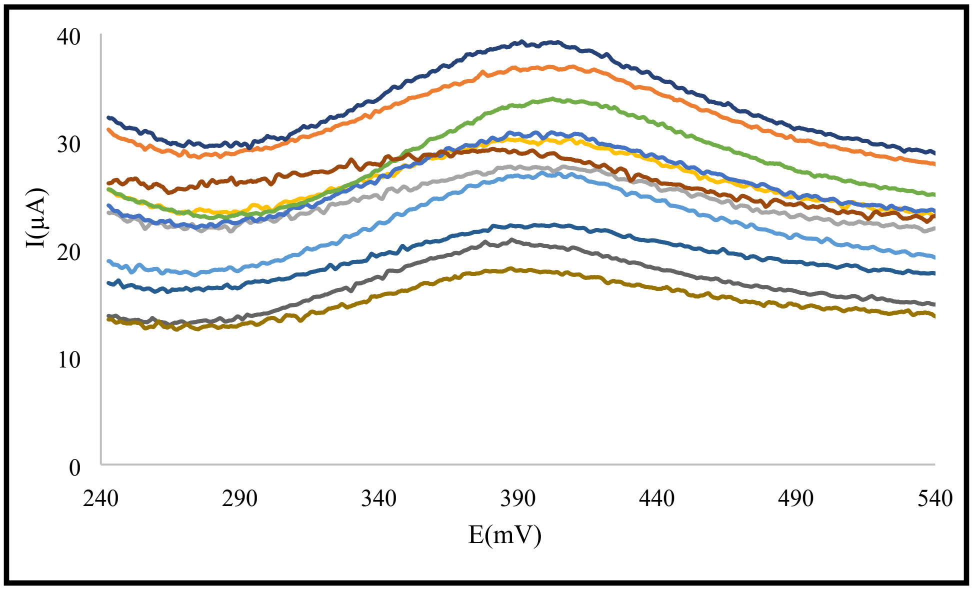

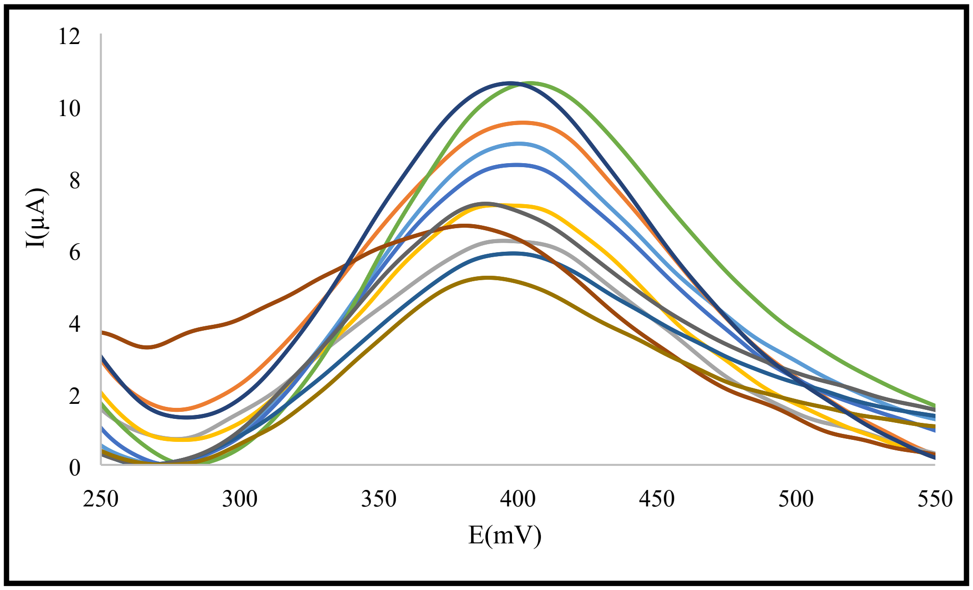

Fig. S8. SWAS voltammograms of 4-NP (100ppm) at the Ni-MOF74/ $Fe_3O_4/SiO_2/NH_2$/ β-CD / GCE surface in the PBS (0.1M, pH= 9.40) at different scan rate (27.5-112mvs^{-1}) and different duration time (11-240 s), Pulse=500 mV. Experimental data (a) and Pretreated Data (b)

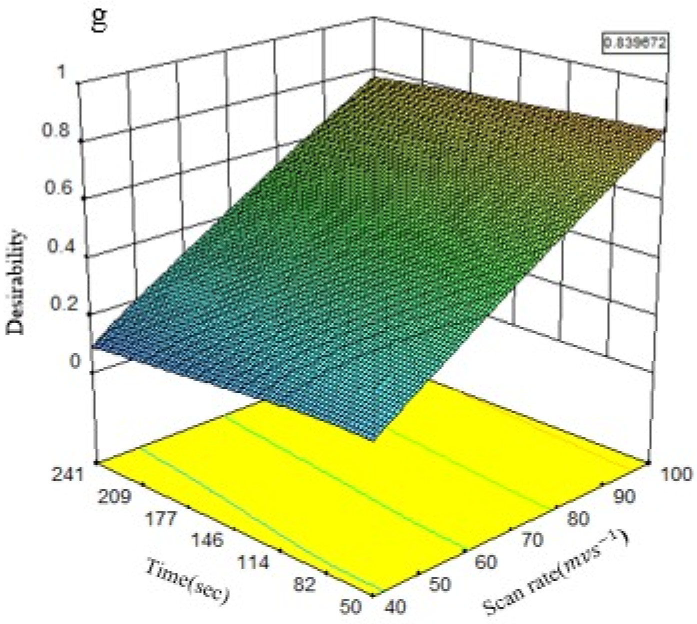

Fig.S9. 3-D desirability plot vs. scan rate and duration time to optimize 2-NP and 4-NP oxidation process

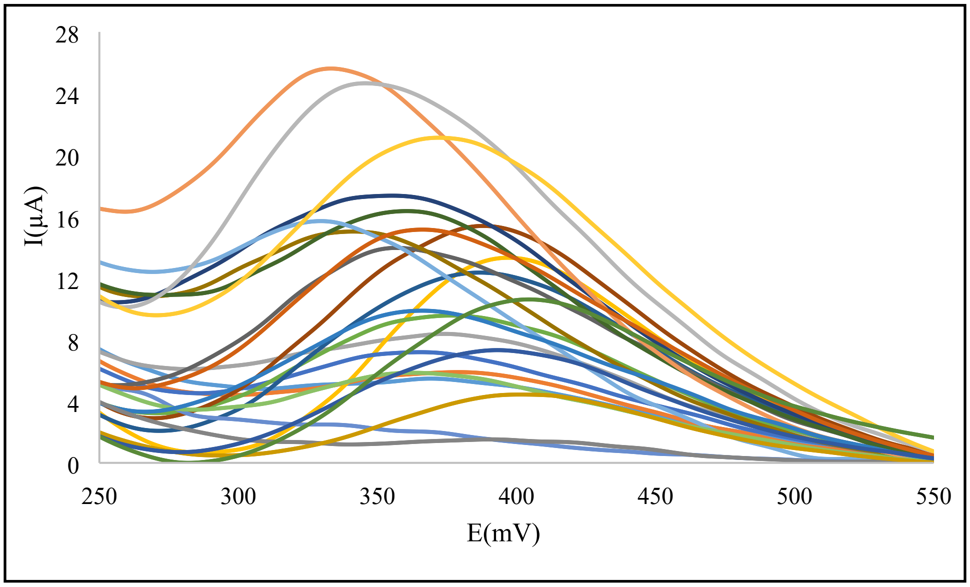

Fig. S10. SWAS voltammograms of 2-NP and 4NP at the Ni-MOF74/ $Fe_3O_4/SiO_2/NH_2$/ β-CD / GCE surface in the PBS (0.1M, pH= 9.40) at scan rate 100mvs^{-1} and duration time 50s, Pulse=500 mv, for to total data set

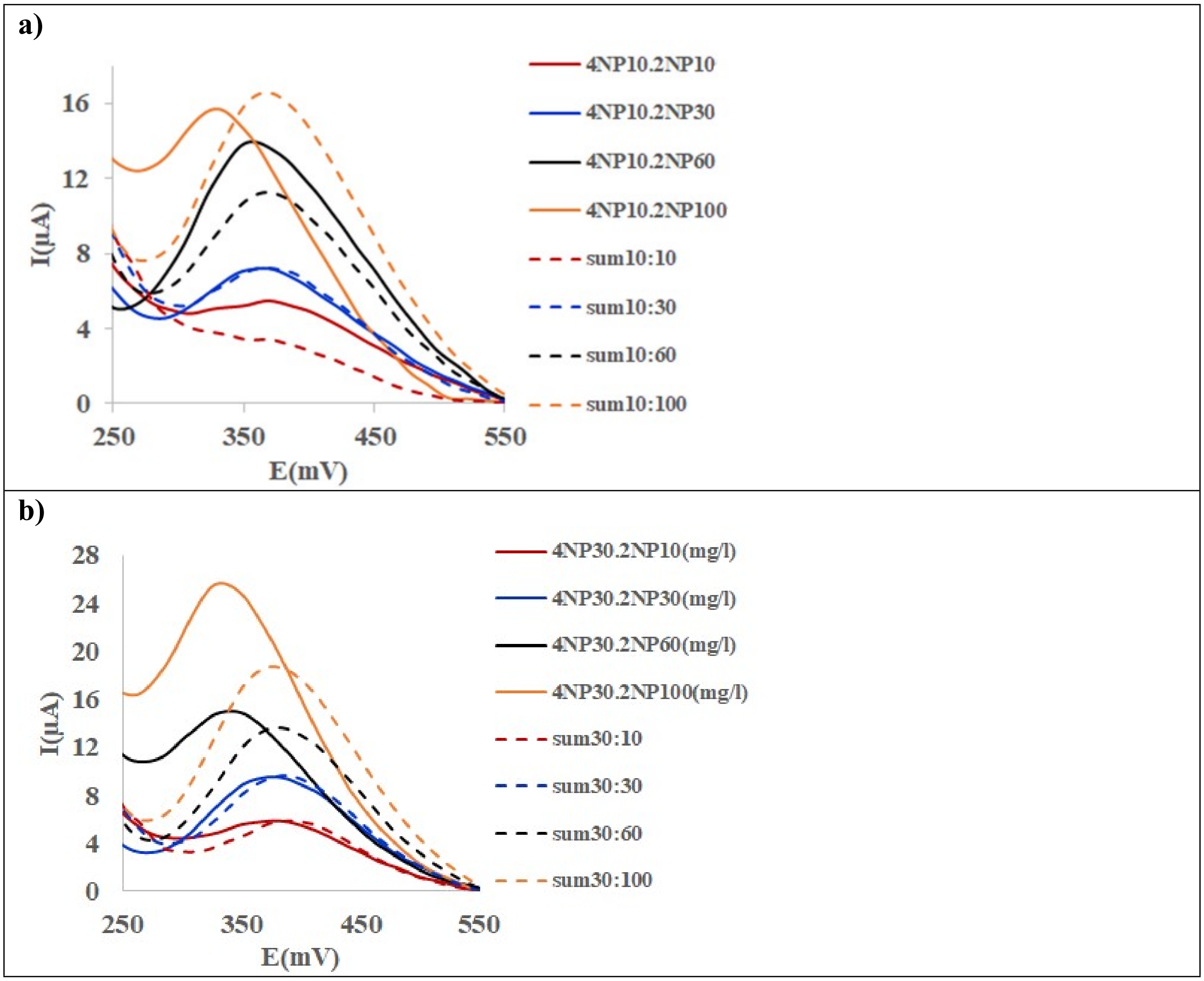

Fig.S11. SWAS voltammograms of (a): 4-NP (10ppm) in 2-NP (10-100ppm). (b): 4-NP (30ppm) in 2-NP (10-100ppm).

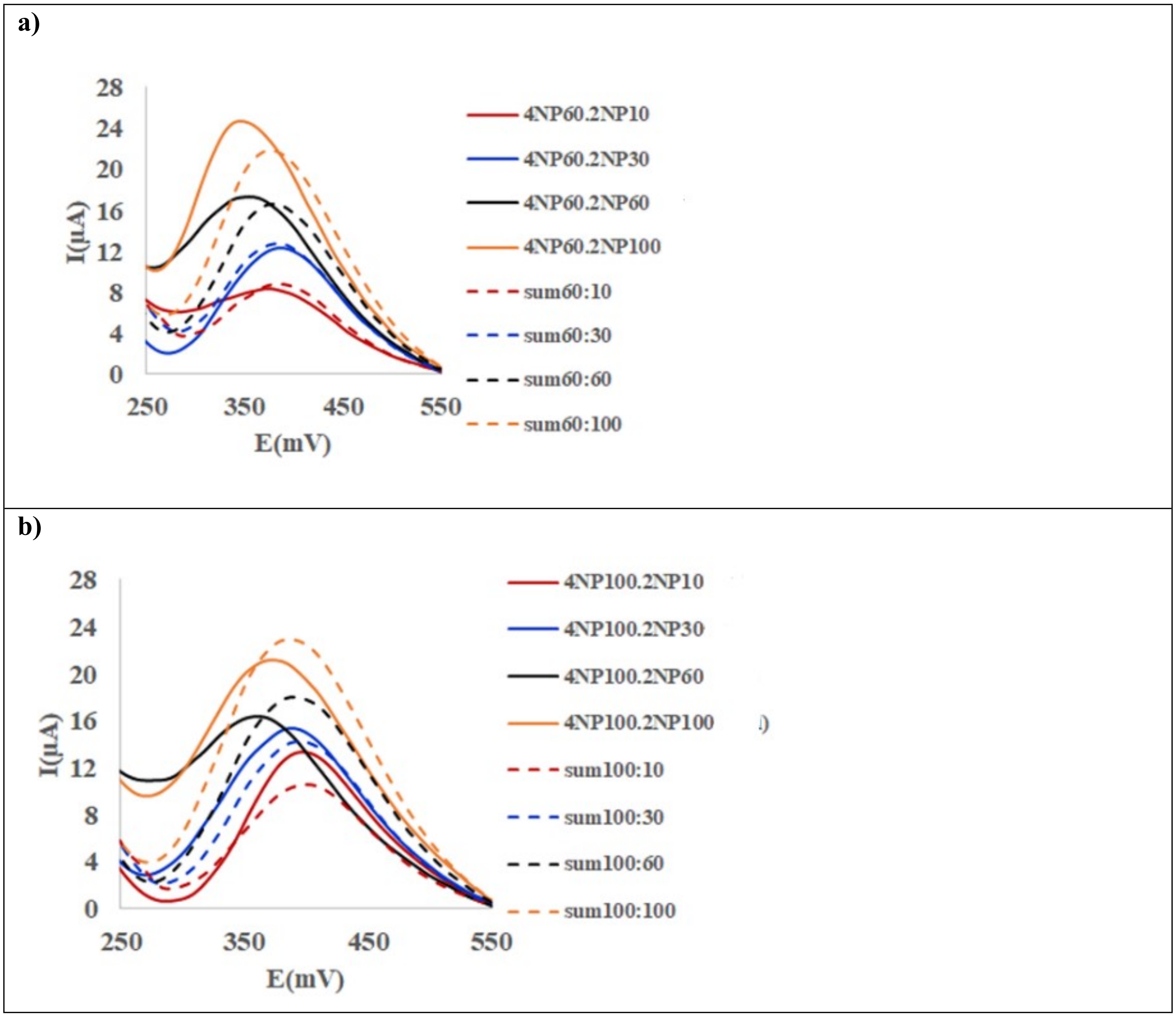

Fig. S12. SWAS voltammograms of (a): 4-NP (60ppm) in 2-NP (10-100ppm). (b): 4-NP (100ppm) in 2-NP (10-100ppm), at the Ni-MOF74/ $Fe_3O_4/SiO_2/NH_2$/ β-CD / GCE surface in the PBS (0.1M, pH= 9.40) at scan rate 100mvs^{-1} and duration time 50s, Pulse=500 mv.

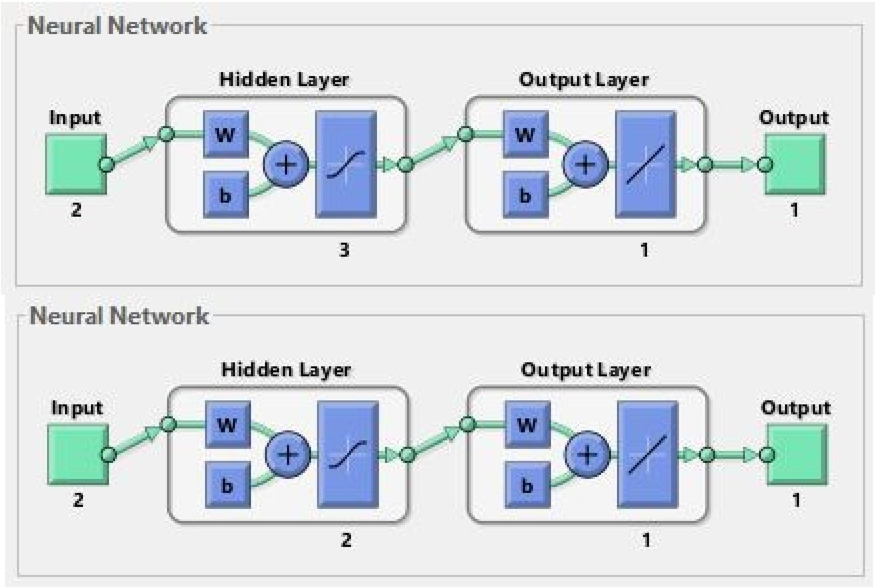

Fig.S13. the ANN's architecture for 2-NP and 4-NP

Table S1. Analysis of variance (ANOVA) result for the model of 2-NP10

Source	Sum of Squares	df	Mean Square	F-value	p-value Prob> F	
Model	0.044	2	0.022	19.84	0.0008	significant
A-A	0.038	1	0.038	34.35	0.0004	
A^2	6.146×10^{-3}	1	6.146×10^{-3}	5.51	0.0468	
Residual	8.920×10^{-3}	8	1.115×10^{-3}			
Lack of Fit	5.004×10^{-3}	6	8.340×10^{-4}	0.43	0.8234	not significant
Pure Error	3.916×10^{-3}	2	1.958×10^{-3}			
Cor Total	0.053	10				

Table S2. Analysis of variance (ANOVA) result for the model of 2-NP30

Source	Sum of Squares	df	Mean Square	F-value		p-value Prob> F
Model	0.018	5	3.623×10^{-3}	19.19	0.0028	significant
A-A	0.018	1	0.018	94.65	0.0002	
B-B	5.241×10^{-6}	1	5.241×10^{-6}	0.028	0.8742	
AB	1.438×10^{-4}	1	1.438×10^{-4}	0.76	0.4228	
A^2	4.569×10^{-6}	1	4.569×10^{-6}	0.024	0.8825	
B^2	9.272×10^{-5}	1	9.272×10^{-5}	0.49	0.5147	
Residual	9.438×10^{-4}	5	1.888×10^{-4}			
Lack of Fit	7.992×10^{-4}	3	2.664×10^{-4}	3.68	0.2209	not significant
Pure Error	1.447×10^{-4}	2	7.234×10^{-5}			
Cor Total	0.019	10				

Table S3. Analysis of variance (ANOVA) result for the model of 2-N100

Source	Sum of Squares	df	Mean Square	F-value		p-value Prob> F
Model	9.212×10^{-3}	5	1.842×10^{-3}	28.66	0.0011	significant
A-A	8.146×10^{-3}	1	8.146×10^{-3}	126.70	< 0.0001	
B-B	6.753×10^{-4}	1	6.753×10^{-4}	10.50	0.0229	
AB	2.196×10^{-4}	1	2.196×10^{-4}	3.42	0.1238	
A^2	1.590×10^{-4}	1	1.590×10^{-4}	2.47	0.1766	
B^2	2.459×10^{-5}	1	2.459×10^{-5}	0.38	0.5634	
Residual	3.215×10^{-4}	5	6.429×10^{-5}			
Lack of Fit	7.387×10^{-5}	3	2.462×10^{-5}	0.20	0.8898	not significant
Pure Error	2.476×10^{-4}	2	1.238×10^{-4}			
Cor Total	9.534×10^{-3}	10				

Table S4. Analysis of variance (ANOVA) result for the model of 4-NP10

Source	Sum of Squares	df	Mean Square	F-value	p-value Prob> F	
Model	6.803×10^{-3}	1	6.803×10^{-3}	9.03	0.0148	significant
A-A	6.803×10^{-3}	1	6.803×10^{-3}	9.03	0.0148	
Residual	6.777×10^{-3}	9	7.530×10^{-4}			
Lack of Fit	1.340×10^{-3}	7	1.915×10^{-4}	0.070	0.9966	not significant
Pure Error	5.437×10^{-3}	2	2.718×10^{-3}			
Cor Total	0.014	10				

Table S5. Analysis of variance (ANOVA) result for the model of 4-NP30

Source	Sum of Squares	df	Mean Square	F-value		p-value Prob> F
Model	9.798×10^{-3}	5	1.960×10^{-3}	7.29	0.0240	significant
A-A	8.421×10^{-3}	1	8.421×10^{-3}	31.34	0.0025	
B-B	2.104×10^{-5}	1	2.104×10^{-5}	0.078	0.7908	
AB	3.956×10^{-5}	1	3.956×10^{-5}	0.15	0.7170	
A^2	4.274×10^{-4}	1	4.274×10^{-4}	1.59	0.2629	
B^2	4.839×10^{-4}	1	4.839×10^{-4}	1.80	0.2373	
Residual	1.344×10^{-3}	5	2.687×10^{-4}			
Lack of Fit	4.447×10^{-4}	3	1.482×10^{-4}	0.33	0.8096	not significant
Pure Error	8.990×10^{-4}	2	4.495×10^{-4}			
Cor Total	0.011	10				

Table S6. Analysis of variance (ANOVA) result for the model of 4-NP60

Source	Sum of Squares	df	Mean Square	F-value		p-value Prob> F
Model	4.614×10^{-3}	5	9.228×10^{-4}	9.50	0.0137	significant
A-A	4.393×10^{-3}	1	4.393×10^{-3}	45.21	0.0011	
B-B	1.727×10^{-4}	1	1.727×10^{-4}	1.78	0.2400	
AB	3.600×10^{-5}	1	3.600×10^{-5}	0.37	0.5693	
A^2	5.095×10^{-6}	1	5.095×10^{-6}	0.052	0.8280	
B^2	3.508×10^{-6}	1	3.508×10^{-6}	0.036	0.8568	
Residual	4.859×10^{-4}	5	9.717×10^{-5}			
Lack of Fit	1.235×10^{-4}	3	4.117×10^{-5}	0.23	0.8718	not significant
Pure Error	3.624×10^{-4}	2	1.812×10^{-4}			
Cor Total	5.100×10^{-3}	10				

Table S7. Analysis of variance (ANOVA) result for the model of 4-N100

Source	Sum of Squares	df	Mean Square	F-value	p-value Prob> F	
Model	3.046×10^{-3}	5	6.093×10^{-4}	6.80	0.0277	significant
A-A	2.517×10^{-3}	1	2.517×10^{-3}	28.10	0.0032	
B-B	1.726×10^{-4}	1	1.726×10^{-4}	1.93	0.2237	
AB	4.851×10^{-5}	1	4.851×10^{-5}	0.54	0.4948	
A^2	7.720×10^{-5}	1	7.720×10^{-5}	0.86	0.3958	
B^2	2.881×10^{-4}	1	2.881×10^{-4}	3.22	0.1329	
Residual	4.479×10^{-4}	5	8.957×10^{-5}			
Lack of Fit	2.044×10^{-4}	3	6.815×10^{-5}	0.56	0.6916	not significant
Pure Error	2.434×10^{-4}	2	1.217×10^{-4}			
Cor Total	3.494×10^{-3}	10				

Table S8. The network parameters in the MATLAB toolbox.

**Topology**	2 inputs, 1 output and 1 hidden layer with 2 neurons 2-NP: ($2\times2\times1$), 3 neurons 4-NP ($2\times3\times1$),
**Data**	Training set: 24 randomly selected data structures
	Test set: 3 randomly selected data structures
	Validation set: 3 randomly selected data structures
**Beginning function**	Log–sigmoid
**Training algorithm**	Levenberge–Marquardt
**Loss function conditions**	Minimum MSE
**Stopping conditions**	The network stops in one of three way:<br>Validation check > 10<br>Minimum gradient < 10^{-7}<br>Momentum speed > 10^{10}
